# RNAseK is vital for epithelial function in *Drosophila melanogaster*

**DOI:** 10.1242/jeb.252529

**Published:** 2026-09-03

**Authors:** Chandan Maurya, Andrew D. Gillen, Shannon Keenan, Julian A. T. Dow

**Affiliations:** ^1^ School of Molecular Biosciences, University of Glasgow, Glasgow G12 8QQ, Scotland, UK; ^2^ Shared Research Facilities, University of Glasgow, Glasgow G12 8QQ, Scotland, UK

**Keywords:** V-ATPase, Proton pumps, Epitheliome, Malpighian tubules

## Abstract

The transporting epithelial tissues comprising the *Drosophila melanogaster* alimentary and renal systems are known to share a common set of enriched genes sometimes referred to as the ‘epitheliome’, reflecting their shared transport functions. Core amongst these genes are the *vha* genes, which encode subunits of the large vacuolar-type ATPase (V-ATPase) proton pump complex. However, many of the non-vha components of the epitheliome remain broadly uncharacterised. Here, we explored the role of *RNAseK*, a gene identified during unbiased epithelial screens in *Drosophila* whose function within insects is not yet known, though evidence from mammalian systems suggests a role in supporting proton pump activity. We demonstrate computationally that *RNAseK* is strongly conserved across evolutionary history, and that expression is regulated by the highly epithelium-specific dCLEAR motif. Seeking to understand why epithelial expression is so emphasised, we assayed the effects of *RNAseK* knockdown in different epithelia throughout the fly. Across hindgut, midgut and Malpighian tubules, we note profound defects in gross tissue morphology, transport activity and fly survival. Mechanistically, RNAseK co-localises apically with the V-ATPase subunit Vha55, and *RNAseK* knockdown phenotypes overlap with those caused by V-ATPase perturbation. These findings are consistent with a role for RNAseK in acidification-dependent epithelial transport and membrane homeostasis, although direct effects on V-ATPase biochemical activity or on other epithelial transport and junctional pathways remain to be tested.

## Teaser

**Summary:** RNAseK is enriched throughout, and required in, *Drosophila melanogaster* epithelial tissues. Molecular and evolutionary evidence supports a possible role in acidification-dependent epithelial transport and membrane homeostasis.

## INTRODUCTION

The fruit fly *Drosophila melanogaster* provides a prime opportunity for interrogating tissue function in the context of a rich experimental genetic framework. Aside from providing direct insights into fly biology, *Drosophila* tissues share a high degree of functional homology with human tissues, so studying tissue function in the fly can be an efficient strategy to study human disease (Verheyen, 2022; Pandey and Nichols, 2011). *Drosophila* research can also provide a window into insect biology as possibly the most well-studied species within Hexapoda.

*Drosophila*’s alimentary canal is composed of foregut, midgut and hindgut, as well as salivary glands and Malpighian tubules. These tissues play an important role in nutrient transport, either in fluid secretion [salivary gland (Maruyama and Andrew, 2012) and Malpighian tubules (Beyenbach et al., 2010)] or in nutrient absorption [midgut and hindgut (Colombani and Andersen, 2020)]. For this reason, these tissues may be referred to collectively as the ‘transporting epithelia’. Previous efforts into identifying key genetic determinants of the transporting epithelia, termed the ‘epitheliome’, have identified several genes whose expression appears to be closely linked to epithelial specification (Chintapalli et al., 2013; Gillen et al., 2025).

Core amongst epitheliome components are several subunits of the vacuolar-type ATPase (V-ATPase) holoenzyme. V-ATPases are membrane-bound proton pumps composed of 15 subunits, which can be divided into a cytoplasmic V1 region and a membrane-bound V0 region (Dow et al., 1997). V-ATPases are critical for many aspects of cell biology, including maintenance of electrostatic gradients (Dow et al., 1997; D'Silva et al., 2017) and maintaining appropriate pH balance in cells and vesicles (Dulac et al., 2021; Yan et al., 2009). As a result of these diverse functions, V-ATPase activity is ubiquitous, and not limited to epithelial tissues (Dulac et al., 2021; Jain et al., 2024).

That said, the 15 canonical V-ATPase subunits are encoded by 33 *vha* genes within *Drosophila*, allowing numerous theoretically possible configurations of the overall holoenzyme structure (Allan et al., 2005). Given this fact, it is striking that epithelial tissues consistently and preferentially express 15 *vha* genes comprising a distinct V-ATPase holoenzyme from non-epithelial tissues (Chintapalli et al., 2013, 2007; Gillen et al., 2025), an expression profile which seems to be co-ordinated by the transcription factor MITF (Bouché et al., 2016).

Intriguingly, though, there is evidence that this view of epithelial V-ATPase function remains incomplete. In particular, evidence from other species has recently uncovered roles for homologues of the highly conserved transmembrane protein RNAseK in supporting the function of V-ATPase (Makar et al., 2024; Perreira et al., 2015). Hypothesis-free analysis of *Drosophila* epitheliome components has also identified RNAseK as extremely closely linked to epithelial function (Gillen et al., 2025).

RNAseK, or ribonuclease kappa, is relatively poorly understood within *Drosophila melanogaster.* Localised to the plasma membrane, RNAseK may be utilised by viruses to enter *Drosophila* cells (Hackett et al., 2015), but no direct evidence of functionality within *Drosophila* has yet been found. That said, evidence from humans, who possess a closely related *RNAseK* orthologue, provides a clear picture of a ribonuclease (Economopoulou et al., 2007) which also acts as a V-ATPase associated factor required for functionality of the holoenzyme (Makar et al., 2024). Structural analyses of mammalian V-ATPase place RNAseK/RNASEK within the V0 sector of the holoenzyme, while recent functional work indicates that RNAseK-dependent phenotypes can involve lysosomal hydrolase delivery and autophagosome degradation, rather than being interpreted solely as a direct global acidification defect (Abbas et al., 2020; Makar et al., 2024).

Based on our previous screen and evidence from related systems, we hypothesised that RNAseK may support epithelial V-ATPase-associated pathways in *Drosophila*, thereby contributing to pan-epithelial function. Here, we provide an overview of transcriptomic and evolutionary data supporting this possibility, demonstrate a role for RNAseK throughout *Drosophila* epithelia and show evidence linking this role to acidification-dependent epithelial function.

## MATERIALS AND METHODS

### Data utilisation

*Drosophila melanogaster* annotation information were obtained from FlyBase (https://flybase.org/; release FB2025_03). Specifically, a list of *Drosophila* transcription factors was compiled based on FlyBase gene group FBgg0000745, and gene identifier mapping information was derived from fbgn_fbtr_fbpp_expanded_fb_2025_03.tsv.gz.

*Drosophila melanogaster* transcription factor binding motifs were obtained by combining the interactome reported by Shokri et al. (2019) with those available through CisBP (Weirauch et al., 2014).

### Regulatory motif analysis

Analysis of known regulatory motifs shared within a gene set was carried out using i-*cis*Target (Imrichová et al., 2015) (https://gbiomed.kuleuven.be/apps/lcb/i-cisTarget/index.php). The settings were as follows: full motif collection; i-*cis*Target motif database v.6.0; for *Drosophila melanogaster* (dm6) with gene annotation FlyBase 6.02. The search region was defined as 5 kb upstream of each gene's transcriptional start site to the end of the transcript, with a 0.4 minimum fraction of overlap with identified motifs. Normalised enrichment score thresholding was set to 3 (the default), based on enrichment calculated within each motif data separately and an ROC threshold of 0.01 for area-under-curve analysis.

*De novo* motif identification was carried out using FindMotifs, part of the Hypergeometric Optimization of Motif EnRichment (HOMER) suite (Heinz et al., 2010) (http://homer.ucsd.edu/homer/, v.5.1, 7-16-2024). Enrichment of sequence motifs was analysed over the HOMER *Drosophila melanogaster* promoter set. The search region was defined as 2 kb upstream of each gene's transcriptional start site to 2 kb downstream – the maximal search region permitted within HOMER. Other settings for this analysis were as follows: Repeats were masked; Motifs up to 14 bp searched; all other settings at default.

Additionally, new software was developed to check for motif occurrence and enrichment at the same time. Using BLAMM for motif searching (Fostier, 2020), all genes (± a defined window size) are checked for occurrences of a given motif. Specific genomic co-ordinates for each occurrence are returned and, if multiple genes of interest are specified, motif frequency is compared with general motif occurrence rates using χ^2^ testing. This software is freely available on GitHub (https://github.com/AndrewDGillen/MotifScanning) and figshare (https://doi.org/10.6084/m9.figshare.31037749).

### Multiple sequence alignment

Multiple sequence alignment was run using Muscle5 (Edgar, 2022) via the EBI online portal (https://www.ebi.ac.uk/jdispatcher/msa/muscle5) using default settings. This was run three times, to analyse conservation separately for RNAseK protein, cDNA and gene sequences.

*RNAseK* orthologues were identified using OrthoDB (Tegenfeldt et al., 2025). Orthologues were as follows: *Anopheles gambiae* ENSAGBG00005004689; *Aedes aegypti* AAEL002492; *Schistocerca gregaria* XM_049992438.1; *Bombyx mori* LOC732881 and LOC101747017; *Tribolium castaneum* TC008797 and TC006752; *Adalia bipunctata* ENSABPT00005007386.1; *Apis melliflera* LOC725572; *Danio rerio* ENSDARG00000104458 and ENSDARG00000069461; *Mus musculus* ENSMUSG00000093989; *Homo sapiens* ribonucleaseK.

### Fly stocks and husbandry

*Drosophila* lines of the desired genotypes were maintained on standard Glasgow *Drosophila* diet (Dornan et al., 2023) under controlled conditions of 45–55% relative humidity, a 12 h light/dark cycle, and a temperature of 24±1°C in a Sanyo incubator unless otherwise specified. For GAL4/UAS system experiments with the tub Gal80^TS^ transgene, parental (control) and F1 (experimental) flies were initially reared at the permissive temperature of 18°C. After eclosion, experimental F1 progeny were shifted to the restrictive temperature of 29°C to inactivate Gal80^TS^ and permit gene expression; simultaneously, control flies were also transferred to 29°C to ensure age-matched comparisons.

The following fly lines were acquired from the Bloomington *Drosophila* Stock Centre (Bloomington, IN, USA; Table S1):

y v; P{y[+t7.7]=CaryP}attP2 (BL #36303); y sc[] v sev; P{y[+t7.7] v[+t1.8]=TRiP.HMS01529}attP2/TM3, Sb (BL #35779); y w[]; PBac{y[+mDint2] w[+mC]=UAS-RNASEK.HA}VK00033 (BL #94316); w; P{w[+mC]=PTT-GA}VhaSFD[G00259]/CyO (BL #6840); P{w[+m*]=sens-GAL4.B}1, y w[] (BL #42740).

Myo31 df Gal4; tub Gal80^TS^ (Adam Dobson Lab, University of Glasgow, Scotland, UK); da Gal4; tub Gal80^TS^ (Ilan Davies Lab, University of Glasgow, Scotland, UK); Irk-2 Gal4/Cyo; UAS-GFP/TM6c (B. Denholm Lab, University of Edinburgh, Scotland, UK) were obtained from collaborative laboratories.

Other experimental lines were generated and maintained in-house: w*; CapaR-GAL4 (Terhzaz et al., 2012), w*; UAS mCD8::GFP; CapaR-GAL4’, ‘C-724 GAL4 (Sözen et al., 1997), and introgressed line w; C-724 GAL4; UAS-mGFP (Dornan et al., 2023).

### Immunocytochemistry and Phalloidin staining

Dissected tissues from 9 day old adult *Drosophila* were collected in Schneider's *Drosophila* medium (Gibco, Thermo Fisher Scientific, cat. no. 20012-019) at room temperature, then fixed in freshly prepared 4% PFA in 1× phosphate-buffered saline (PBS) for 20 min to ensure cellular structure preservation. After fixation, the tissues were permeabilised by three successive 15 min washes with PBS containing 0.1% Triton X-100 (PBST; Sigma-Aldrich, cat. no. 93443-100ML, 10% stock in H_2_O). For blocking, tissues were incubated for 2 h at room temperature in a solution of 5% Normal Goat Serum and 0.5% Triton X-100 in 1× PBS (Gibco, Ref. 20012-019).

For immunostaining, tissues were incubated overnight at 4°C in blocking buffer containing mouse monoclonal anti-Dlg1 (1:50, 4F3, DSHB) and rabbit polyclonal anti-RNAseK (1:1000, Geneosphere Biotechnologies) antibody. The RNAseK antibody was custom-generated in host rabbit against the peptide EDLPLEEEYHSLEDC, coupled to keyhole limpet haemocyanin (KLH), and affinity purified with ELISA verification (absorption for positives was >2-fold over pre-immune serum).

Following the primary antibody step, tissues were washed three times for 15 min each in 0.1% PBST. For secondary antibody detection, Alexa Fluor 546-conjugated anti-rabbit IgG (cat. no. A-11035, Thermo Fisher Scientific) and Alexa Fluor 633-conjugated anti-mouse IgG (cat. no. A-21052, Thermo Fisher Scientific) were each diluted 1:500 and incubated with samples for 2 h at 37°C. After secondary antibody incubation, tissues were washed and then treated with DAPI (1 μg ml^−1^, Thermo Fisher Scientific, cat. no. D21490) prior to mounting on Polysine slides (VWR International, cat. no. 631-0107) with antifade medium, followed by imaging using a Zeiss LSM 880 confocal microscope (Carl Zeiss).

For Phalloidin staining, tissues were dissected, fixed and permeabilised in the same way but incubated with Phalloidin-Atto 550 (Sigma-Aldrich, cat no. 19083) at 1:1000 dilution for 15 min at room temperature to stain F-actin. After staining, tissues were washed three times in 0.1% PBST, followed by incubation in DAPI (1 μg ml^−1^) for 10 min and three additional washes with 0.1% PBST. These samples were also mounted on Polysine slides with antifade medium as above and imaged on the Zeiss LSM 880 confocal microscope. Confocal image stacks were analysed with ImageJ (ImageJ 2.16.0), and representative *z*-projections were prepared for figure assembly in Microsoft PowerPoint (v.2405).

### Stellate cell counting

For quantification of stellate cells in control and *RNAseK* downregulated flies, C-724 transgenic flies recombined with mGFP were crossed with UAS control and *RNAseK* knockdown flies. The Malpighian tubules were dissected from 9 day old adult female flies in 1× PBS and fixed in 4% PFA for 10 min at room temperature. Fixed tubules were mounted on glass slides using antifade mounting medium, and stellate cells were manually counted under a fluorescence microscope. Counts from anterior and posterior tubules were recorded separately. The raw data were analysed using GraphPad Prism 10 (v.10.2.3), and statistical significance was determined using multiple non-parametric comparisons.

### RNA isolation, cDNA synthesis and qPCR

RNA was isolated from 50 pairs of freshly dissected adult *Drosophila* tubules using QIAzol Lysis Reagent (Qiagen Sciences USA, 50 ml) followed by purification with the miRNeasy Mini Kit (cat. no. 217004, Qiagen Sciences USA). The final RNA prep was resuspended in nuclease-free dH_2_O provided with the kit. For cDNA synthesis, 300 ng of total RNA was used in a SuperScript II RT reaction (cat. no. 18064-022, Thermo Fisher Scientific), according to the manufacturer's instructions. qPCR was carried out using the QuantiNova SYBR Green PCR Kit (cat. no. 208054, 500 reactions, Qiagen) on a StepOnePlus Real-Time PCR system (Applied Biosystems), with gene-specific primers. All primers were synthesised by Integrated DNA Technologies (IDT). Primer sequences were as follows: *Rpl32* (TTTCGCTTCTGGTTTCCGG; CTTGCGCCATTTGTGCGA); *dPrestin* (AGTTTGCCAAGGTGCGTTC; CAGGAGTACGGCTTCAGTCC); *SPoCk* (GCATTCGTCATTTTTGCTGCG; AGGGTAAGGCCCGTCTGTT); *Dh44-R2* (GGTCGATAGCCTGGATGACG; GAATCGAACGAGCTGGGACA); *Crystallin* (CAGCACCAGATTTGAGCGTC; GTGAGAGAGGGGCGAAACTC); *RNAseK* (TGTGGCCCGAAACTATCACTT; GCAGTTGTAGGCATTCTGATTGT); *vha68-2* (GAACGCGAAGACACAGCAAA; CAGTTACGCCAGAGGTCTCC).

Primer specificity was confirmed by running PCR products on agarose gels using cDNA as the template – with gDNA used as a negative control on the same gel – and by checking for single melt curve peaks in qPCR. For each 96-well plate (cat. no.4346907), 5-fold dilution series standard curves were generated in duplicate alongside sample amplifications to ensure amplification met the MIQE standard 90–110% efficiency threshold (Bustin et al., 2009). Results from qPCR analysis were normalised to Rpl32 expression, providing the gene/Rpl32 expression ratio per sample. Final data were plotted using GraphPad Prism 10 (v.10.2.3).

### Starvation and desiccation assays using DAM5H monitors

All experiments were performed using the DAM5H *Drosophila* Activity Monitor (TriKinetics Inc.) under standard laboratory conditions (24±1°C; ambient humidity maintained within the DAM incubator). Flies aged 9 days (both sexes) were briefly anaesthetised and loaded individually into 65 mm glass tubes. In the starvation assay, tubes were prepared with approximately 1 cm of 1% agar at one end for humidity, sealed with a rubber plug, and flies were introduced from the opposite end, which was sealed with cotton to maintain aeration; activity was then monitored in the DAM5H for up to 6 days based on optimised female survival. For desiccation, flies were placed in empty tubes sealed with cotton at both ends and monitored for 4 days. After completion, data were extracted from the DAM system, compiled in Microsoft Excel (v.2405) and analysed in GraphPad Prism 10 (v.10.2.3) to identify outliers and compare groups using an unpaired non-parametric Mann–Whitney test for rank comparisons. All experiments were carried out in biological triplicate, and final results were drawn from compiled replicates.

### Midgut pH assays and gut length measurement

*m*-Cresol purple (cat. no. 857890-5G, Sigma-Aldrich) indicator dye was mixed into melted Standard Glasgow *Drosophila* diet containing 1% kaolin (cat. no. K7375-500G, Sigma-Aldrich), cooled to 60°C (final concentration 0.3% w/v), then swirled and allowed it to reach room temperature.

Adult female flies, 9 days old, of the appropriate genotype were placed on the dye-mixed food for overnight feeding. The next day, midguts were dissected in Schneider's *Drosophila* medium (cat. no. 21720-024, Thermo Fisher Scientific), and immediate micrographs were taken with a Zeiss Stemi 508 stereomicroscope equipped with an Axiocam 105 colour camera, as pH remains stable for only a few minutes after dissection. Images were acquired using Zeiss Zen 3.9 software.

For gut length measurements, individual gut images were opened in Zeiss Zen 3.9 software and the length was traced and measured using the spline tool. Recorded lengths were analysed in GraphPad Prism 10 (v.10.2.3) using an unpaired Mann–Whitney test for rank comparisons.

### Ramsay fluid secretion assay

Fluid secretion assays using *Drosophila* Malpighian tubules (MTs) were performed following established protocols (Halberg et al., 2015; Dow et al., 1994). Anterior tubules were dissected in ice-cold Schneider's medium and placed in a 9 μl drop containing a 1:1 mix of Schneider's medium and *Drosophila* saline with trace amaranth dye (cat. no. A1016, Sigma-Aldrich). After acclimation, baseline secretion rates were measured for each tubule at 10 min intervals over a 30 min period. Subsequently, 1 μl of CAPA neuropeptide (10^−6^ mol l^−1^; SOLASTA Bio, Glasgow, Scotland, UK) was added to the drop, and fluid secretion rates were measured every 10 min for an additional 30 min. A diuretic effect was confirmed if secretion increased after CAPA application compared with basal levels.

This experiment was performed using Malpighian tubules from 9 day old female flies in three independent biological replicates. For final analysis, data from all replicates were combined, and statistical significance was determined using two-way ANOVA with the Greenhouse–Geisser correction, a family-wise alpha threshold, and comparison with a 95% confidence interval. Graphs were generated in GraphPad Prism 10 (v.10.2.3).

### Bafilomycin feeding and gut analysis

Canton-S flies were sorted into control and treatment groups from day 1 of age and reared until day 9. Control flies were maintained on 5% sucrose solution for the entire 9 day experimental period. For the treatment group, flies were placed in separate vials containing 200 μl of 5% sucrose solution supplemented with 50 μmol l^−1^ bafilomycin A1 (cat. no: 54645, Cell Signaling Technologies). Flies were sorted into control and treatment groups from day 1 of age and reared until day 9. The feeding solution was replaced daily, and all vials were kept in a dark chamber throughout the assay to prevent light-induced degradation of bafilomycin A1 and to ensure its stability.

To analyse gut pH partitioning following treatment, flies were given overnight access to food containing *m*-Cresol purple dye (cat. no. 857890-5G, Sigma-Aldrich) and 1% kaolin (cat. no. K7375-500G, Sigma-Aldrich), following the midgut pH measurement protocol. Immediately after feeding, midguts were dissected and imaged using a Zeiss Stemi 508 stereomicroscope with an Axiocam 105 colour camera, and images as well as gut length measurements were processed using Zeiss Zen 3.9 software for both bafilomycin-treated and control flies.

### Fly mass and water content measurements

To measure wet body mass, 20 9 day old *Drosophila* from each replicate (*n*=9; consisting of three biological replicates with three technical replicates per biological replicate, and both sexes included) were anaesthetised and transferred into pre-weighed 1.5 ml tubes (cat. no. 616 201, Greiner Bio-One). The flies were weighed immediately after transfer using a GR-202 analytical balance (A&D Instruments, Abingdon, UK). For dry body mass measurement, flies were first killed by freezing at −80°C for 20 min, then dried at 65°C for 72 h in a Hybridiser HB-1D (Techne). After reaching room temperature post-drying, flies were weighed again using the same analytical balance. Water content was calculated based on the difference between wet and dry mass. Percentage water content, along with all mass data, was statistically analysed using an unpaired Mann–Whitney test for rank comparisons in GraphPad Prism 10 (v.10.2.3).

### Smurf intestinal permeability assay

For assessing intestinal barrier integrity, the Smurf assay was performed following the protocol adapted from Martins et al. (2018). Standard melted fly food was cooled to approximately 60°C, and FD&C Blue Dye No.1 (cat no. 861146, Sigma-Aldrich) was added at a final concentration of 2.5% w/v (2.5 g per 100 ml). The food was gently mixed, swirled to ensure even dye distribution, and allowed to cool fully to room temperature. Adult male and female flies of the desired genotypes were transferred to freshly prepared dyed food and maintained for 14 h of feeding at standard laboratory conditions.

Flies were subsequently transferred to fresh, non-dyed standard media for immediate visualisation. Scoring was performed via visual inspection for blue coloration throughout the body – the so-called Smurf phenotype – which indicates intestinal barrier dysfunction and leakage of the non-absorbable blue dye beyond the digestive tract. The flies were classified into Non-Smurf, Uncertain, Light Smurf and Smurf categories, and the proportions were converted to percentages. The graph was plotted using GraphPad Prism 10 (v.10.2.3).

### *Drosophila* feeding assay (pipette tip)

To conduct the *Drosophila* feeding assay, individual 200 μl pipette tips (cat. no. S1120-8810, TipOne Pipette Tips, Starlab) were first weighed using a GR-202 analytical balance (A&D Instruments) to record their initial empty mass (M1). Each tip was then filled with 30 μl of freshly prepared 5% sucrose solution, and the mass was recorded again (M2). Ten adult flies (9 days old) were introduced into each assay vial, and a loaded pipette tip was inserted vertically to ensure flies could access the sucrose solution freely. The vials were maintained at 25°C in a Sanyo incubator for 24 h, allowing the flies to feed. After 24 h, the pipette tip was removed and weighed to determine the final mass (M3). The volume of sucrose consumed was calculated by subtracting M3 from M2 and converting this mass change to microlitres. Feeding data were analysed statistically using a Mann–Whitney test, in GraphPad Prism 10 (v.10.2.3).

### Embryo fixation and visualisation

Embryos (20–22 h) were dechorionated by immersing them in freshly prepared 50% sodium hypochlorite (bleach) solution for 2 min with gentle agitation, effectively removing the outer chorion layer. After dechorionation, embryos were thoroughly washed with double distilled water to eliminate any remaining bleach. For permeabilisation and fixation, embryos were vigorously shaken in a 1:1 mixture of 4% PFA fixative and *n*-heptane (cat. no. GPS9716, apc pure) for 30 s, ensuring the fixative could penetrate the vitelline membrane efficiently. The embryos were then washed three times with 0.1% PBST, each wash lasting 3 min, to remove any residual fixatives and permeabilising agents. Finally, embryos were mounted onto slides and visualised under polarised light using a Zeiss Stemi 508 stereomicroscope equipped with an Axiocam 105 colour camera. Micrographs were acquired using Zeiss Zen 3.9 software for subsequent analysis.

### Alizarin Red staining

Malpighian tubules from 9 day old flies of the desired genotypes were dissected in 1× PBS solution and immediately fixed in 4% PFA for 15 min to preserve tissue architecture. The fixed tubules were then rinsed three times for 5 min each in 0.1% PBST to ensure thorough permeabilisation. Staining was performed by incubating the tubules in Alizarin Red S (cat. no. A5533-25G, Sigma-Aldrich) at a concentration of 20 mg ml^−1^ (filtered twice using Whatman™ cat. no. 1001 125) dissolved in 0.1% PBST for 4 min.

After staining, the tubules were destained and rinsed three times in 0.1% PBST for 5 min each on a shaker to remove excess dye. The stained tubules were then mounted and imaged under transmitted polarised light using a Zeiss Stemi 508 stereomicroscope equipped with an Axiocam 105 colour camera. Images were acquired and analysed using Zeiss Zen 3.9 software for presentation.

### Abdominal width determination

Flies of the desired genotype and age were positioned laterally for imaging using a Zeiss Stemi 508 stereomicroscope equipped with an Axiocam 105 colour camera. The acquired image was opened in Zeiss Zen 3.9 software, where abdominal width was measured. The resulting values were statistically analysed using an unpaired Mann–Whitney test for rank comparisons, in GraphPad Prism 10 (v.10.2.3), allowing for robust comparison of abdominal size across genotypes or experimental groups.

## RESULTS

### RNAseK follows epithelial transcriptional programmes

**Fig. 1.**
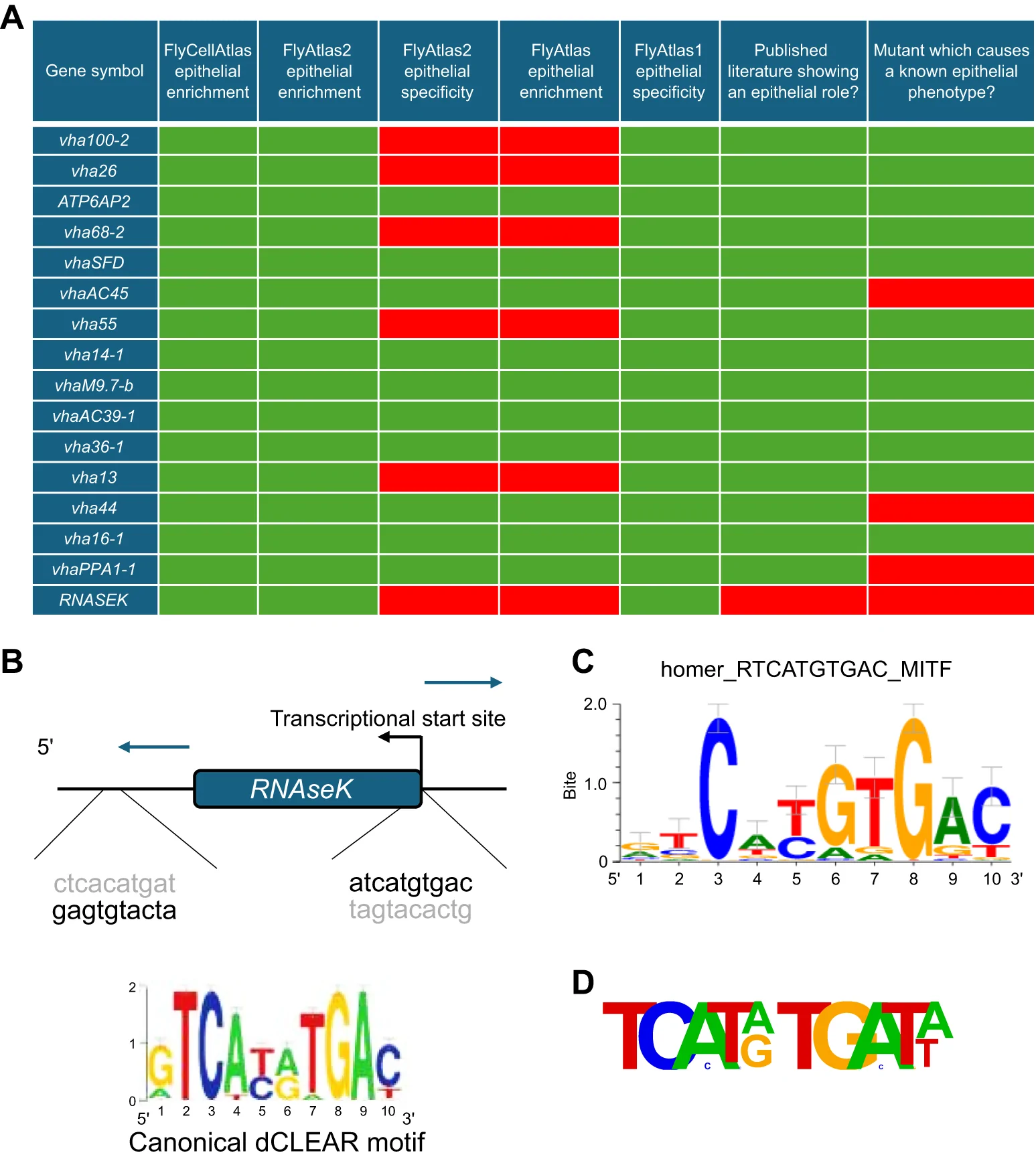
**RNAseK expression is V-ATPase-like in epithelial tissues.** (A) A summary of the published transcriptomic evidence supporting an epithelial role for RNAseK, based on meta-analysis performed using DIGITtally (Gillen et al., 2025). Boxes are shaded green if a gene fulfils the criteria and red if it does not. (B) Predicted dCLEAR motifs surrounding the *RNAseK* gene (Bouché et al., 2016). Arrows indicate the directionality of the motif, with the reverse complement shown in grey. (C,D) The most significantly enriched known regulatory motif associated with RNAseK and the 15 epithelial V-ATPase subunits as calculated with (C) i-*cis*Target, searching the sequence from 5 kb upstream of each gene's transcriptional start site to the end of the transcribed region, and (D) HOMER, searching the sequence from 2 kb upstream of each transcriptional start site to 2 kb downstream.

Hypothesis-free analysis of large, publicly available data have demonstrated that RNAseK broadly follows a pattern of enrichment across epithelial tissues within *D. melanogaster* (Cohen et al., 2020). Strikingly, this closely mirrors the evidence supporting epithelial enrichment of V-ATPase subunits (Fig. 1A).

The epithelial pattern of *vha* genes contributing to the epithelial V-ATPase is maintained by the transcriptional regulator MITF, via a motif referred to as dCLEAR (Bouché et al., 2016). To investigate whether this motif could also be responsible for co-ordinating epithelial RNAseK, we developed software to study the motifs around a given gene (available from GitHub: https://github.com/AndrewDGillen/MotifScanning).

Using this software, we identified two putative dCLEAR-like motifs within two kilobases (kb) of the *RNAseK* gene, one overlapping the *RNAseK* transcriptional start site (2R:4188117-4188127) and one within 2 kb of the *RNAsek* end site (2R:4184962-4184972) (Fig. 1B).

To uncover whether the dCLEAR motifs occurring in *RNAseK* and the epithelial *vha* genes are expected at random, we used our software to analyse motif occurrence across these 16 genes, and compared the rate of dCLEAR detection with the rest of the known *D. melanogaster* genes. As previously described (Bouché et al., 2016), all 15 epithelial *vha* genes are within 2 kb of a dCLEAR motif. However, this is true of only 43% of genes globally – a difference which χ^2^ testing confirmed is statistically significant (adjusted *P*<0.0001).

Independently of our targeted analysis, we ran *de novo* motif detection software on our *RNAseK* plus *vha* gene set. Both i-*cis*Target and HOMER motif detection returned very similar motifs associated with this gene set (Fig. 1C), which bore striking similarity to the known dCLEAR motif. As such, there is strong evidence that RNAseK, as with the epithelial V-ATPase subunits, is regulated by MITF within *Drosophila* epithelial tissues.

### RNAseK is strongly conserved across evolutionary history

**Fig. 2.**
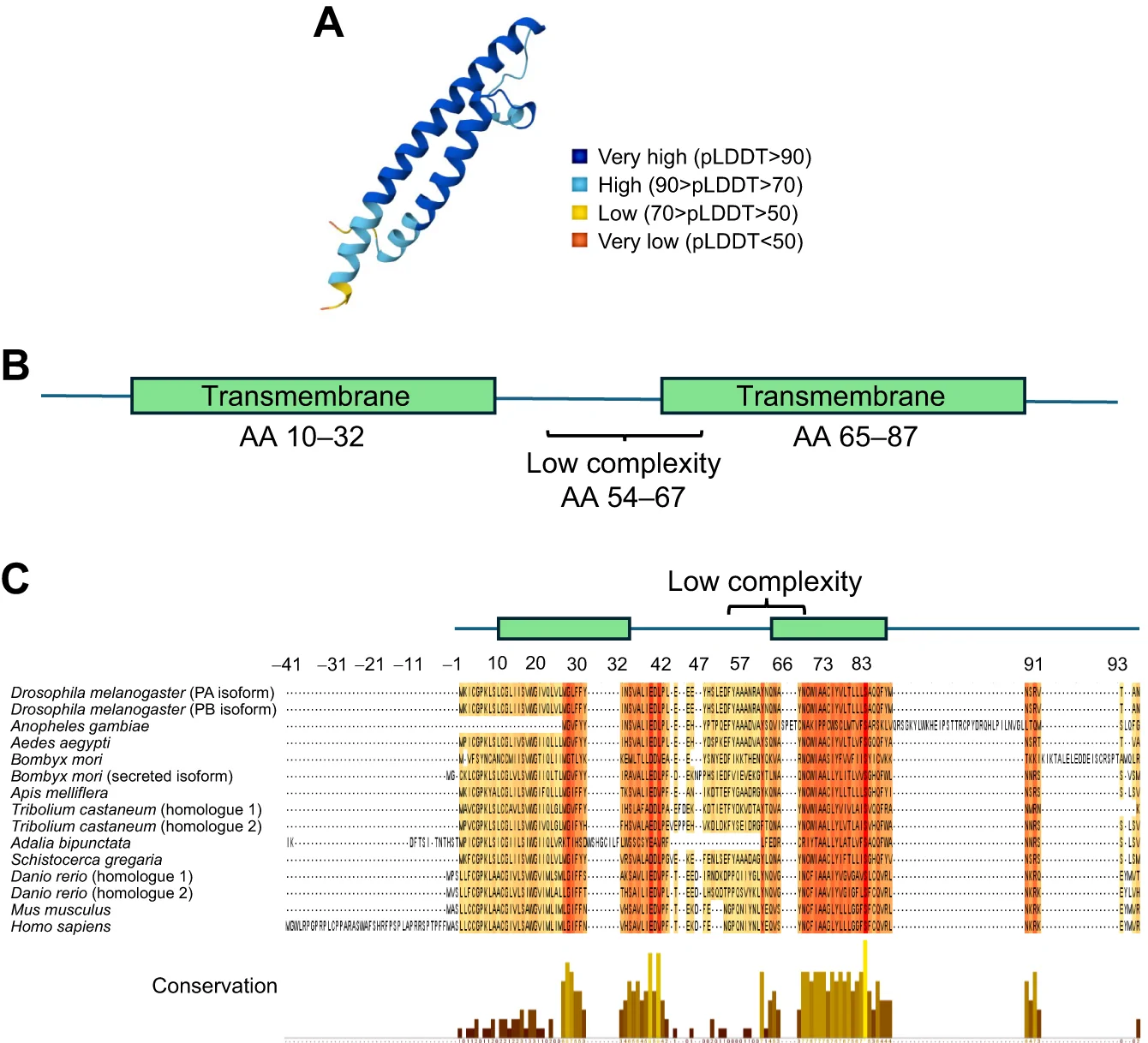
**RNAseK structure is highly conserved.** (A) The 3D structure of RNAseK, as predicted by AlphaFold (Jumper et al., 2021). The colour scheme for by-residue confidence is shown (pLDDT, predicted local distance difference test). (B) Diagrammatic representation of domains found within RNAseK, as predicted by SMART (Letunic et al., 2021). (C) Conservation of the RNAseK protein across *Drosophila melanogaster* isoforms and other select species.

Given this evidence of a shared transcriptional programme and the wealth of evidence that RNAseK is a V-ATPase binding partner in non-fruit fly species (Makar et al., 2024; Perreira et al., 2015), we hypothesised that this activity could be conserved across evolutionary history. As such, we sought to explore conservation of RNAseK homologues as defined by OrthoDB (Tegenfeldt et al., 2025) from *Drosophila*, non-fly insects and other model organisms (Fig. 2).

The structure of RNAseK as predicted by AlphaFold (Jumper et al., 2021) is shown in Fig. 2A, along with a diagrammatic representation of the domains contained within the protein, based on SMART predictions (Letunic et al., 2021) (Fig. 2B). Critically, this predicts three major structural domains – two transmembrane domains and a low-complexity region.

Within this context, the conservation of RNAseK functionality can be more fully understood. Species of interest were aligned to *D. melanogaster* RNAseK protein sequence, and conservation was recorded across the sequence (Fig. 2C). Notably, conservation was found to be high around the end of the first transmembrane domain, the low-complexity region and the entirety of the second transmembrane domain, and at the end of the protein. Thus, it is entirely conceivable that the function of these domains has been conserved despite enormous evolutionary distance, implying a critical function.

The conservation of RNAseK protein, cDNA and gene sequence globally was measured for all orthologues via multiple sequence alignment (Table 1). Conservation was generally very high amongst other insect species, though protein conservation was higher than cDNA conservation, which was in turn higher than gene conservation. This pattern is the reverse of what has been noted comparing between humans and fruit flies based on the account of Shih et al. (2015), so may imply that the structure of RNAseK protein is under intense selective pressure for conservation.

**Table 1.** Percentage identity of the RNAseK protein, cDNA and gene across other select species

Species	Protein identity	cDNA identity	Gene identity
*Drosophila melanogaster*	100	100	100
*Anopheles gambiae*	47.14	54.13	34.48
*Aedes aegypti*	76.84	63.43	39.06
*Bombyx mori*	56.84	55.67	42.62
*Apis melliflera*	68.43	62.17	46.13
*Tribolium castaneum*	58.95	57.45	37.35
*Adalia bipunctata*	41.03	55.4	38.77
*Schistocerca gregaria*	68.42	55.13	40.83
*Danio rerio*	41.05	42.5	36.92
*Mus musculus*	38.04	41.57	42.99
*Homo sapiens*	38.04	42.3	41.26

In cases where multiple homologues are present in a species, only the results for the homologue with the highest identity to *Drosophila melanogaster* RNAseK are shown. Multiple sequence alignments were carried out using Muscle5 (Edgar, 2022).

Interestingly, though, the sequence divergence of RNAseK is not one-to-one with the course of evolutionary divergence – some homologues show particularly strong or weak conservation. For example, *Schistocerca gregaria* shows high similarity to *D. melanogaster* RNAseK despite being from a wildly divergent taxonomic Order; whilst the mosquito *Anopheles gambiae* shows very limited conservation despite also being a Dipteran species. The evolutionary relationships between studied species, along with phylogenetic trees based on sequence identities, are shown in Fig. S1.

### RNAseK loss disrupts embryonic tubulogenesis and adult feeding ability

Previous functional analyses of vacuolar ATPase (V-ATPase) subunits such as Vha55 established their indispensable role in epithelial polarity and tubular morphogenesis in *Drosophila* (Davies et al., 1996; Dow and Davies, 2001; Allan et al., 2005), prompting assessment of whether the V-ATPase-associated factor RNAseK (Perreira et al., 2015) is similarly essential.

**Fig. 3.**
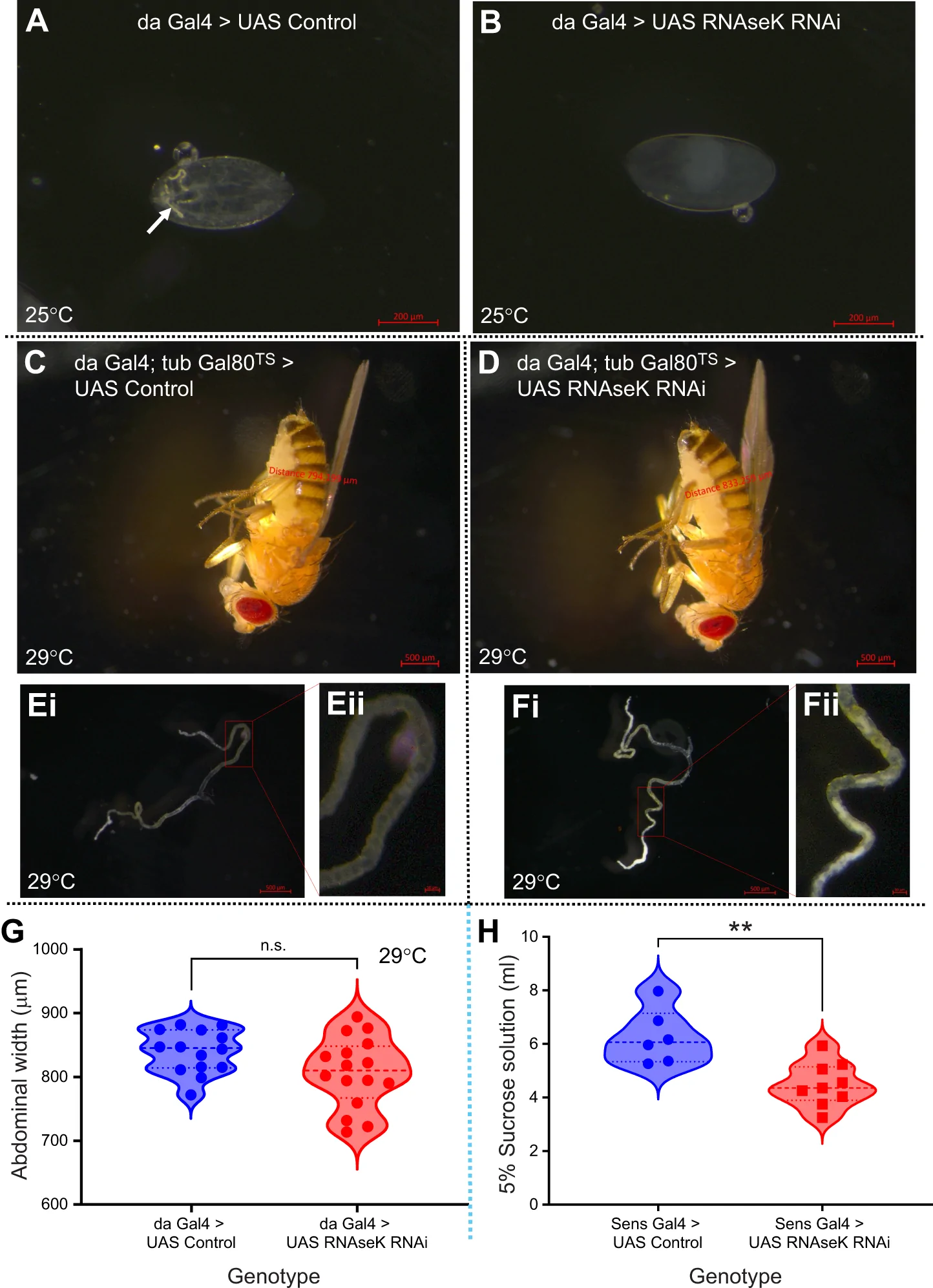
***RNAseK* knockdown affects embryonic development and feeding efficiency.** (A) A da Gal4 > UAS Control embryo imaged at the 20–22 h stage under polarised illumination. Normal Malpighian tubule formation is seen, with crystalline deposits (white arrow). (B) A da Gal4 > UAS RNAseK RNAi embryo at 20–22 h, imaged under polarised light. (C,D) A 9 day old adult female fly, expressing either (C) da Gal4 > UAS Control or (D) da Gal4 > UAS RNAseK RNAi. Flies developed at 18°C until eclosion, then at 29°C. (E,F) Malpighian tubules extracted from 9 day old adult female flies, expressing either (E) da Gal4 > UAS Control or (F) da Gal4 > UAS RNAseK RNAi, at different magnifications. Flies developed at 18°C until eclosion, then at 29°C. (G) Summary of abdominal width data for the above da Gal4 > UAS Control and da Gal4 > UAS RNAseK RNAi adult females. Width from each individually analysed gut is shown, along with a violin indicating the distribution of the data – wider sections indicate higher data density at those values. Thick dashed lines indicate the median value, while thinner lines indicate thresholds for 25% of values and 75% of values. (H) Results of a feeding assay demonstrating a significant decline in food intake in da Gal4 > UAS RNAseK RNAi flies in the salivary gland compared with da Gal4 > UAS Control flies (aged 9 days) (unpaired Mann–Whitney test, **P≤0.01). Scale bars: 200 μm (A,B); 500 μm (C,D,Ei,Fi); 50 μm (Eii,Fii).

Control embryos maintained at 25°C and imaged 20–22 h after egg laying formed well-organised Malpighian tubules under polarised light (Fig. 3A) (Myat, 2005; Denholm, 2013; Cohen et al., 2020), whereas RNAseK deficiency caused the complete absence of tubule formation (Fig. 3B; Fig. S2B), reminiscent of *vha55* or epithelial-polarity mutants showing tubular collapse during embryogenesis (Davies et al., 1996; Wieczorek et al., 2009).

In adults, flies grown at 18°C until eclosion and shifted to 29°C for conditional *RNAseK* knockdown showed normal body (Fig. 3C,D) and tubule morphology (Fig. 3E,F) and unchanged abdominal width (Fig. 3G; Fig. S3A), indicating preserved renal structure. However, feeding assays revealed significantly reduced nutrient intake in RNAseK downregulated adults (Fig. 3H; Fig. S3C), paralleling the loss of proton-pumping efficiency seen in V-ATPase mutants that compromise secretory granule maturation in salivary glands (Farkaš et al., 2015).

### RNAseK regulates multiple critical midgut functions

**Fig. 4.**
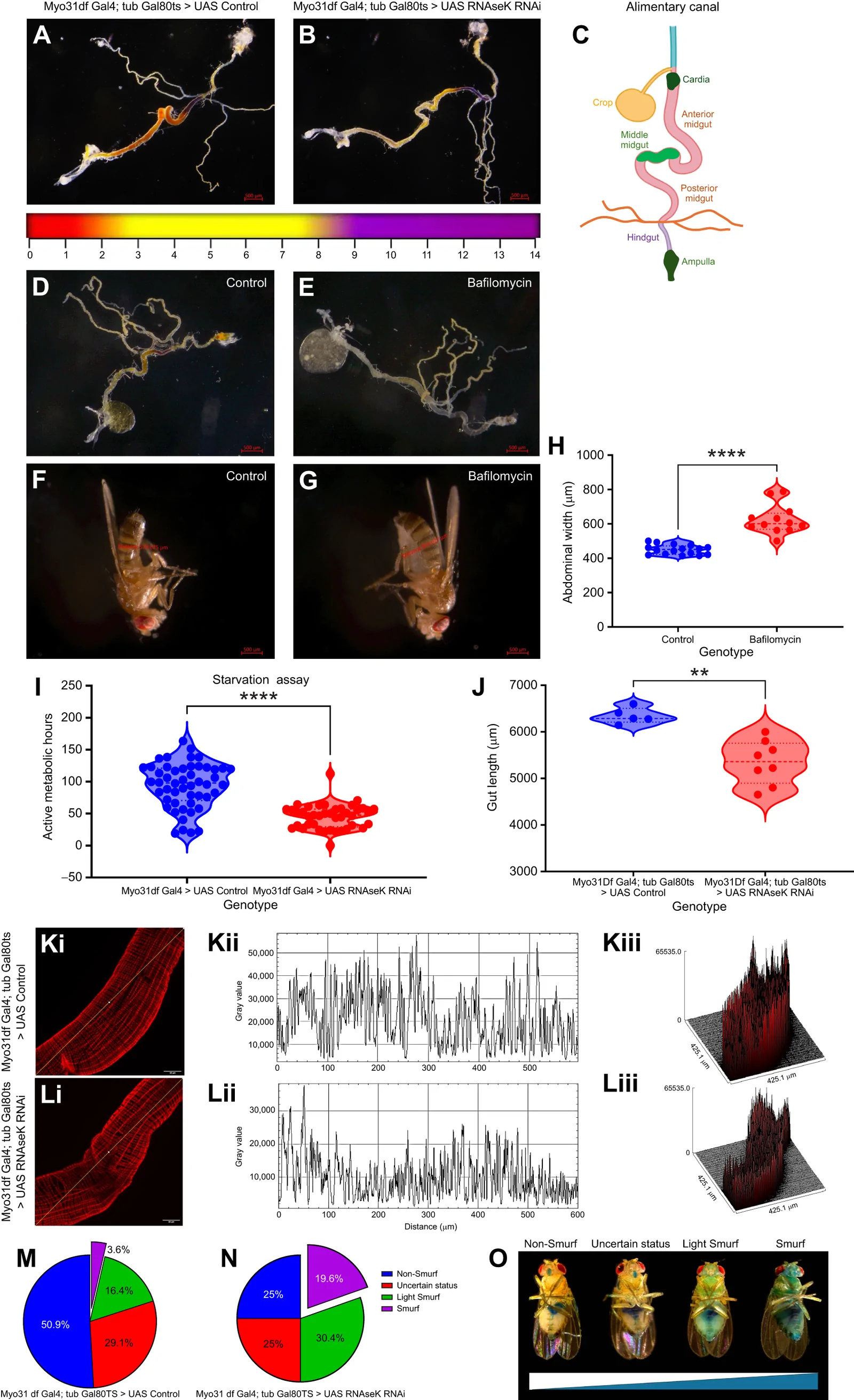
**Midgut *RNAseK* silencing causes extensive digestive system disruptions.** (A,B) Midgut from 9 day old adult female flies, expressing either (A) myo31df Gal4 > UAS Control or (B) myo31df Gal4 > UAS RNAseK RNAi. Flies developed at 18°C until eclosion, then at 29°C. (C) Schematic diagram of the *D. melanogaster* gut. (D,E) Gut morphology of female Canton-S flies fed (D) 5% sugar (control) or (E) 5% sugar with 50 μmol l^−1^ bafilomycin for 9 days. (F,G) Gross morphology of female Canton-S flies fed (F) 5% sugar (control) or (G) 5% sugar with 50 μmol l^−1^ bafilomycin for 9 days. (H) Statistical analysis of abdominal width following bafilomycin feeding to Canton-S flies. (I) Starvation response assayed in control versus RNAseK RNAi flies. (J) Gut length in *RNAseK* knockdown flies relative to controls. (K,L) Phalloidin staining and analysis of midguts from (K) myo31df Gal4 > UAS Control flies and (L) myo31df Gal4 > UAS RNAseK RNAi flies developed at 18°C until eclosion, then reared for 9 days at 29°C: (i) actin organisation, (ii) actin distribution along the marked diagonal line, (iii) surface topology. (M–O) The fly intestinal permeability categorised into Smurf status groups for control (M) and *RNAseK* knockdown (N) flies: Non-Smurf, Uncertain status, Light Smurf and Smurf flies; and the scoring images used for this classification (O). Scale bars: 500 μm (A,B,D–G); 60 μm (Ki,Li). unpaired Mann–Whitney test, **P≤0.01, ****P≤0.0001.

Proton transport by the V-ATPase complex establishes the distinctive midgut pH gradient in *Drosophila*, vital for digestion and copper-cell physiology (Shanbhag and Tripathi, 2005; Strand and Micchelli, 2011; Overend et al., 2016). Control females displayed a typical midgut acidification pattern (Fig. 4A; Fig. S4B), as reported by Dubreuil (2004), while targeted *RNAseK* knockdown resulted in the loss of the characteristic acidic copper-cell zone (Fig. 4B; Fig. S4B). This phenocopies V-ATPase subunit vha100-2, vha100-4 and vha55 loss-of-function mutations (Overend et al., 2016), confirming the dependence of lumenal proton gradient formation on RNAseK.

Treatment with bafilomycin A1, a V-ATPase inhibitor, recapitulated the RNAseK RNAi phenotype of disrupted acidic colouring and abdominal bloating (Fig. 4D–H; Figs S4C and S5C) – consistent with failed V_0_ domain H^+^ translocation (Mauvezin and Neufeld, 2015; Wang et al., 2021). Interestingly, targeted RNAseK downregulation in the hindgut using Irk-2 Gal4 resulted in remarkable localised swelling, presumably indicating functional blockage, probably impacting water excretion and causing the gut to swell (Fig. S5D).

Assays investigating pH gradients in this context revealed a complete loss of the acidic (red) region, which was replaced by a neutral (yellow) profile in these cells (Fig. S5D). This indicates a pronounced disruption of normal pH partitioning, possibly due to neutralisation from abnormal water reflux or unidentified mechanisms. A similar phenotype was seen when the V-ATPase subunit *vha-SFD* was mutated, also resulting in the loss of the characteristic red acidic region from the midgut and the emergence of a neutral pH, suggesting inactivation of the proton pump (Fig. S5E). These findings reinforce that both RNAseK and classic V-ATPase subunits are essential for compartmentalisation of gut pH gradients, and their loss leads to consistent disruption of acid regions through pump inactivation, as supported by Overend et al. (2016) and Dubreuil (2004).

Given the crucial physiological implications, we were particularly interested in the role of RNAseK in the starvation stress response. Consistently, stress assays showed markedly reduced starvation resistance (Fig. 4I) and shortened gut length (Fig. 4J) when RNAseK was genetically downregulated. Notably, this effect is strongly similar to the impaired epithelial maintenance associated with V-ATPase disruption (Perreira et al., 2015; Overend et al., 2016). Phalloidin staining following *RNAseK* knockdown showed disrupted cortical F-actin and cytoskeletal collapse (Fig. 4K,L), consistent with V-ATPase–actin coupling via the WASH/Arp2/3 pathway (Carnell et al., 2011; Lystad, 2025). An intestinal permeability assay also demonstrated that RNAseK RNAi flies exhibited strong Smurf positivity (Fig. 4M–O; Fig. S4A).

### RNAseK is required for the function of both major Malpighian tubule cell types

**Fig. 5.**
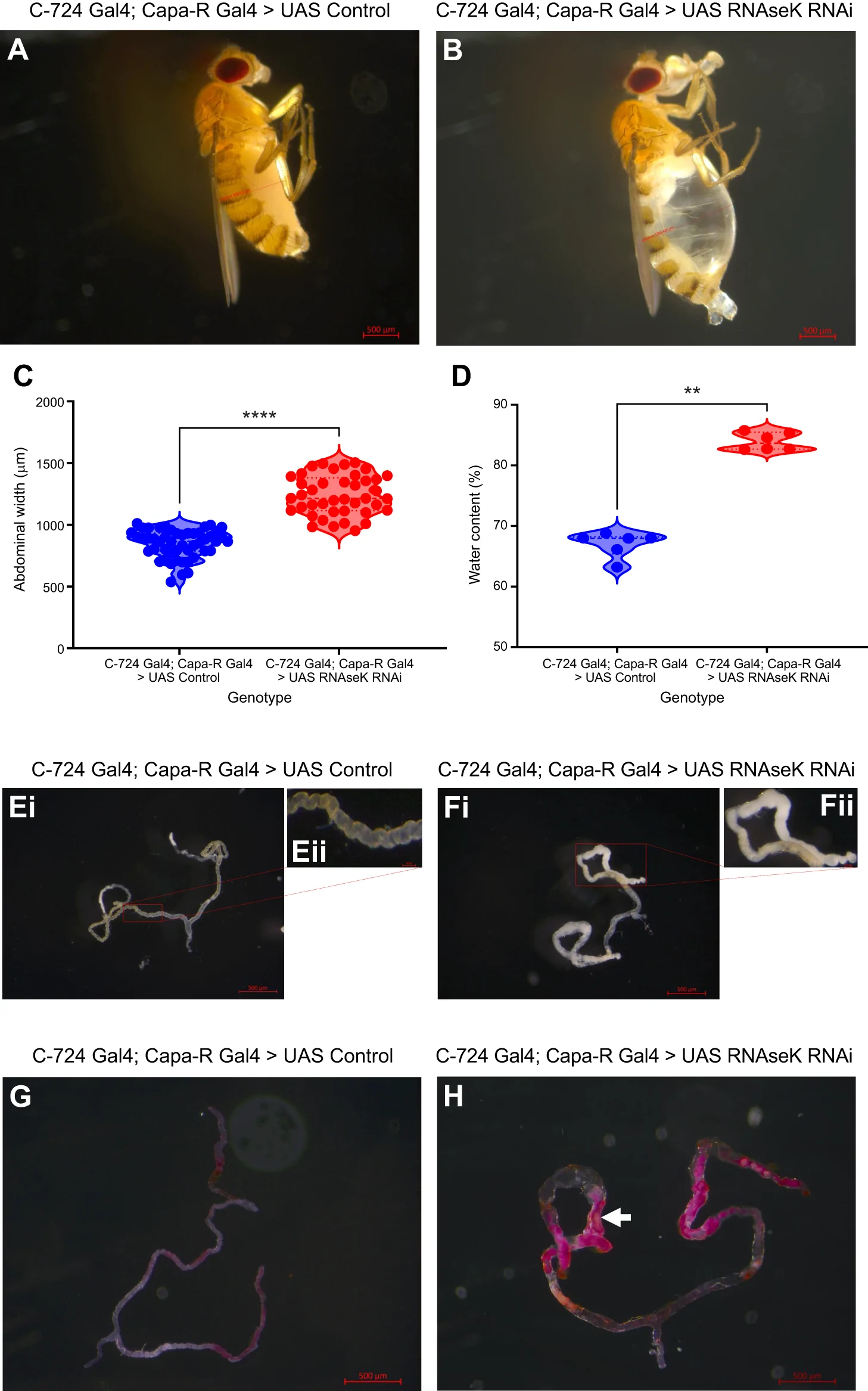
***RNAseK* knockdown across both major cell types (principal and stellate cell) modifies tubule form and body fluid balance in adult flies.** (A,B) Gross morphology of 9 day old adult flies expressing (A) c-724 Gal4 > UAS Control or (B) c-724 Gal4 > UAS RNAseK RNAi. (C) Quantification of abdominal width in control versus *RNAseK* knockdown flies. (D) Comparison of body water content in control versus *RNAseK* knockdown flies. (E,F) Malpighian tubule images from (E) c-724 Gal4 > UAS Control or (F) c-724 Gal4 > UAS RNAseK RNAi flies: (i) the whole Malpighian tubule, (2) a magnified view of the outlined region. (G,H) Alizarin Red-stained Malpighian tubules from (G) c-724 Gal4 > UAS Control or (H) c-724 Gal4 > UAS RNAseK RNAi flies. Scale bars: 500 μm (A,B,Ei,Fi,G,H), 50 μm (Eii,Fii). unpaired Mann–Whitney test, **P≤0.01, ****P≤0.0001.

Malpighian tubules act as V-ATPase-powered analogues to the mammalian renal system, coordinating ion and water transport via spatially distinct secretory and reabsorptive segments (Dow et al., 1994; Dow and Davies, 2001; O'Donnell and Maddrell, 1995). Dual RNAseK depletion in principal and stellate cells caused severe abdominal enlargement (Fig. 5A,B), significantly increased abdominal width (Fig. 5C) and elevated water retention (Fig. 5D), representing systemic osmotic failure comparable to chloride channel silencing (Cabrero et al., 2020). Knockdown tubules showed distorted epithelial architecture under polarised light (Fig. 5E,F) and intraluminal crystalline inclusions confirmed by Alizarin Red staining (Fig. 5G,H; Fig. S6C), consistent with calcium precipitation documented in V-ATPase knockdown models (Allan et al., 2005; Dow et al., 2021).

**Fig. 6.**
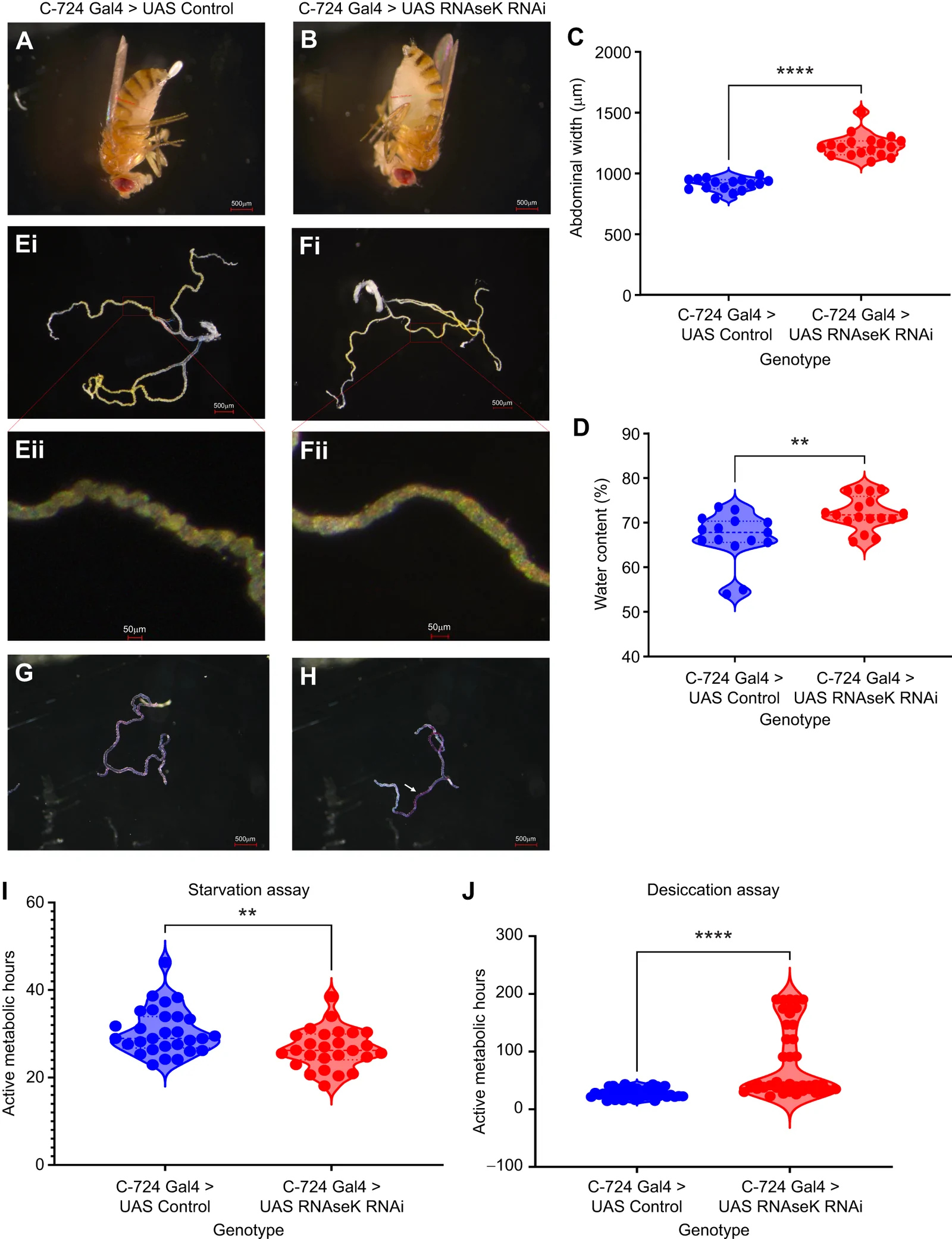
**Stellate cell-specific *RNAseK* knockdown leads to morphological changes with altered physiological resilience and reduced cell abundance in adult flies.** (A,B) Gross morphology of 15 day old flies expressing (A) c-724 Gal4 > UAS Control or (B) c-724 Gal4 > UAS RNAseK RNAi. (C) Quantification of abdominal width across 15 day old flies. (D) Quantification of water content across 15 day old flies. (E,F) Malpighian tubules of 15 day old flies expressing (E) c-724 Gal4 > UAS Control or (F) c-724 Gal4 > UAS RNAseK RNAi: (i) the intact Malpighian tubule, (2) a magnified inset from the same tubule highlighting surface morphology. (G,H) Alizarin Red-stained tubules expressing (G) c-724 Gal4 > UAS Control or (H) c-724 Gal4 > UAS RNAseK RNAi. (I) Starvation response assayed in control versus RNAseK RNAi flies. (J) Desiccation response assayed in control versus RNAseK RNAi flies. Scale bars: 500 μm (A,B,Ei,Fi,G,H); 50 μm (Eii,Fii); 20 μm. Unpaired Mann–Whitney test, **P≤0.01, ****P≤0.0001.

Stellate cell-specific *RNAseK* knockdown (see Fig. S7) resulted in pronounced abdominal swelling (Fig. 6A–C) and elevated water content (Fig. 6D), indicating systemic fluid dysregulation. Microscopy showed severe smoothing of tubule surfaces and depletion of ridged topology (Fig. 6E,F), while Alizarin Red staining identified calcium deposits (Fig. 6G,H; Fig. S8A), signifying ionic imbalance (Dickson, 1965). While the exact mechanistic basis for this disruption of fluid transport caused by stellate cell *RNAseK* knockdown is currently unknown, we hypothesise that it may be related to disruption of V-ATPase-linked acidification (Cabrero et al., 2014; Dow and Davies, 2001). Indeed, both RNAseK and V-ATPase subunits are known to be expressed in stellate cells (Fig. S9; Li et al., 2022), so such a process is eminently possible, though would require further investigation.

Survival assays revealed reduced starvation tolerance upon stellate *RNAseK* knockdown (Fig. 6I) but elevated desiccation resistance (Fig. 6J), phenocopying fluid-retentive adaptations of V-ATPase or ClC mutant flies (Cabrero et al., 2020). Mechanistically, this suggests that RNAseK may regulate coupling between apical acidification and vesicular trafficking, maintaining ClCa and aquaporin localisation required for diuresis (Perreira et al., 2015; Cabrero et al., 2014, 2020).

**Fig. 7.**
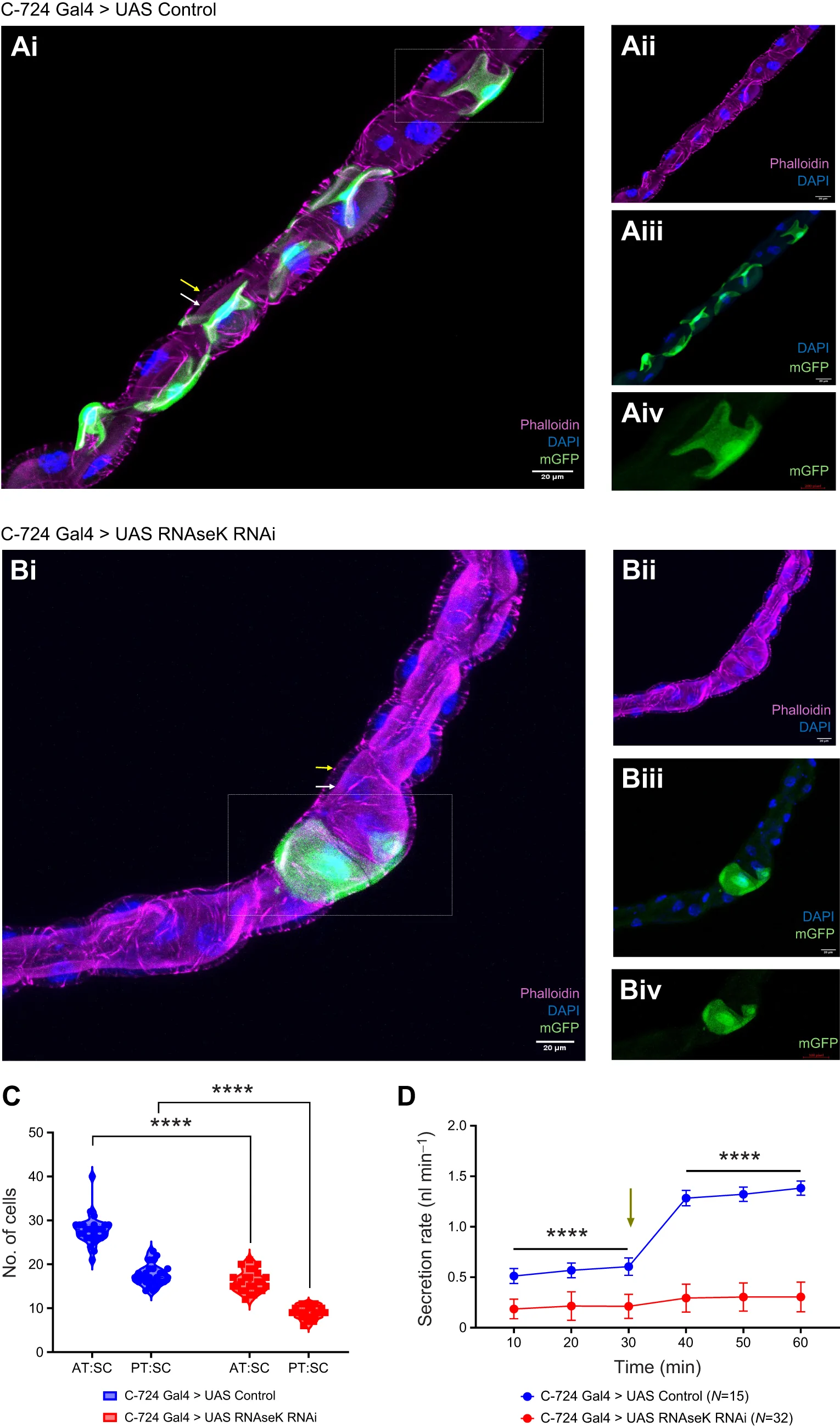
**Stellate cell-specific RNAseK depletion impairs cell morphology, abundance and fluid secretion, assayed in control versus RNAseK RNAi flies.** (A,B) Confocal projections of 15 day old Malpighian tubules from flies expressing (A) c-724 Gal4 > UAS Control or (B) c-724 Gal4 > UAS RNAseK RNAi: (i) Phalloidin (red) labels the actin cytoskeleton, mGFP (green) marks stellate cells and DAPI (blue) stains nuclei; white arrows indicate the apical surface and yellow arrows mark the basal surface; (ii) merged Phalloidin and DAPI channels; (iii) merged mGFP and DAPI channels; (4) magnified view of a single stellate cell indicated by the white arrow in i. (C) Quantification of observed stellate cell numbers. AT, anterior tubule; PT, posterior tubule; SC, stellate cell. (D) Fluid secretion rates in control and RNAseK RNAi tubules under basal and CAPA-stimulated conditions, with the arrow marking the time point of CAPA application. Scale bars: 20 μm (Ai–iv,Bi–iv). Two-way ANOVA with Greenhouse–Geisser correction, ****P≤0.0001, *****P≤0.00001).

Further exploring how RNAseK disruption affects tubule epithelial tissue architecture, extensive imaging of control and *RNAseK*-knockdown flies revealed regularly spaced, actin-rich stellate cells in control tubules (Fig. 7A). In contrast, RNAseK RNAi markedly reduced stellate cell number and distorted their morphology (Fig. 7B; Fig. S10C). Overexpression of *RNAseK* in stellate cells resulted in abnormal stellate cell shape (Fig. S10C), suggesting a role for RNAseK in promoting the proper shaping, spacing and possibly the coordinated integration of stellate cells among principal cells during their mesenchymal-to-epithelial transition in tubulogenesis (Denholm et al., 2003). Quantitative analysis further demonstrated a significant decrease in stellate cell number in both anterior and posterior tubules upon *RNAseK* knockdown (Fig. 7C), reinforcing its importance in stellate cell invagination, distribution and potentially long-term survival. Fluid-secretion measurements showed strong reduction under both unstimulated and CAPA-stimulated conditions (Fig. 7D), indicating compromised neuropeptide-driven diuresis (Davies et al., 2013).

**Fig. 8.**
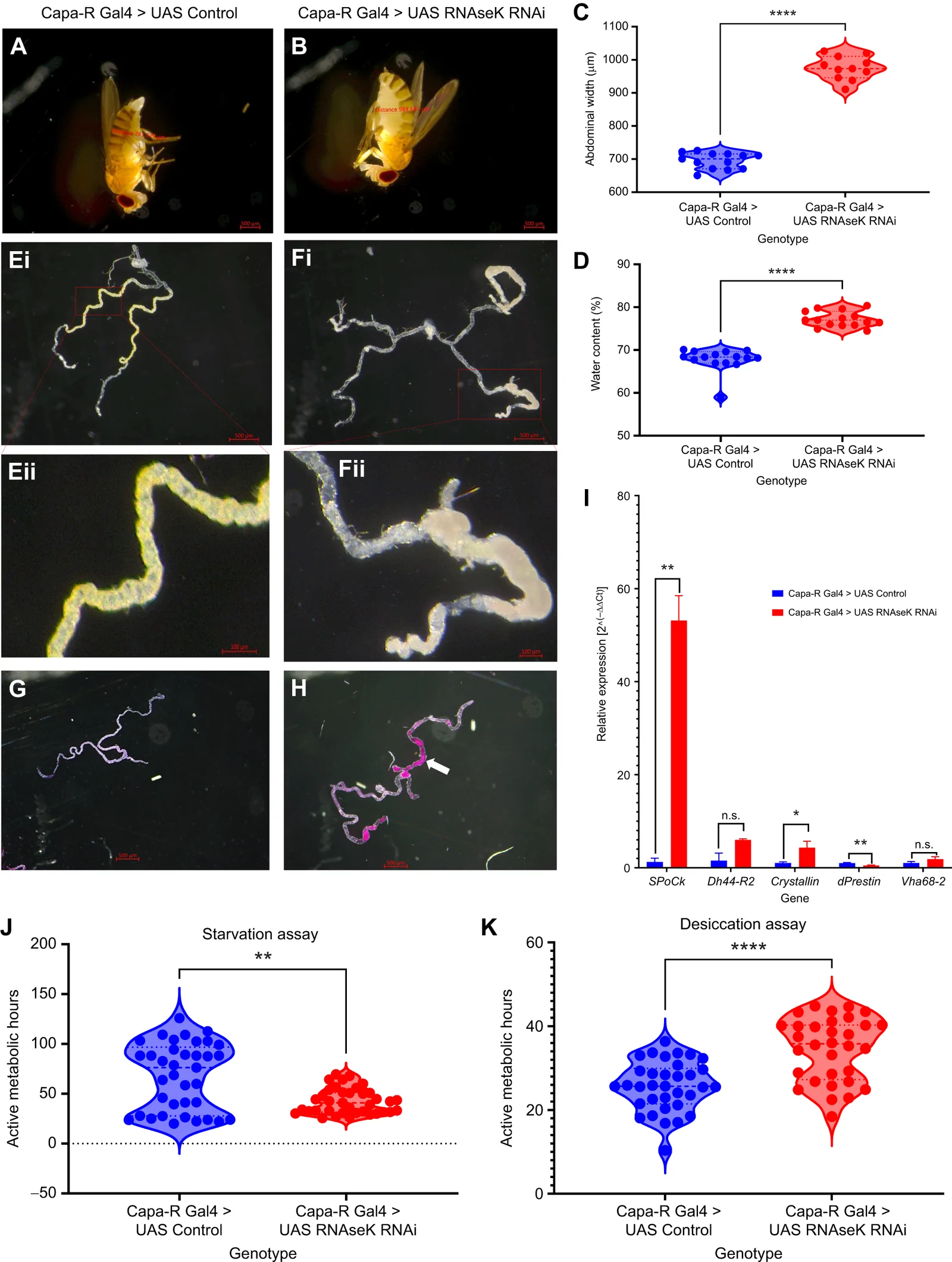
**Principal cell-specific RNAseK suppression remodels tubule architecture and stress behaviour in adult flies.** (A,B) Gross morphology of 9 day old control flies expressing (A) Capa-R Gal4 > UAS Control or (B) Capa-R Gal4 > UAS RNAseK RNAi. (C) Quantitative analysis of observed abdominal width. (D) Quantification of water content across 9 day old flies. (E,F) Malpighian tubule images from (E) Capa-R Gal4 > UAS Control or (F) Capa-R Gal4 >UAS RNAseK RNAi flies: (i) the whole Malpighian tubule, (ii) a magnified view of the outlined region. (G,H) Alizarin Red-stained Malpighian tubules from (G) Capa-R Gal4 > UAS Control or (H) Capa-R Gal4 > UAS RNAseK RNAi. (I) qPCR results showing relative expression of calcium-responsive genes in control versus *RNAseK* knockdown flies. (J) Starvation response assayed in control versus RNAseK RNAi flies. (K) Desiccation response assayed in control versus RNAseK RNAi flies. Scale bars: 500 μm (A,B,Ei,Fi,G,H); 100 μm (Eii,Fii). Unpaired Mann–Whitney test, *P≤0.05, **P≤0.01, ****P≤0.0001.

Principal cell RNAseK RNAi flies displayed abdominal swelling (Fig. 8A–C) and fluid accumulation (Fig. 8D; Fig. S11A–D), accompanied by cystic tubule morphology and white, non-birefringent crystalline deposits (Fig. 8E,F) indicative of calcium phosphate/xanthine crystals (Ghimire, 2019), further supported by Alizarin Red staining (Fig. 8G,H). Transcriptional profiling (Fig. 8I) revealed upregulation of *SPoCk* and *Crystallin*, known calcium-responsive genes (Southall et al., 2006; Chung and Turney, 2017), but no change in *vha68-2*, supporting a post-translational regulatory function of RNAseK.

Functionally, RNAseK-deficient flies exhibited reduced starvation and desiccation tolerance (Fig. 8J,K; Fig. S12B), consistent with metabolic imbalance and impaired water excretion similar to LKR mutants with defective diuresis (Cannell et al., 2016). These results establish RNAseK as a potentially critical regulator of principal cell calcium handling and fluid homeostasis.

### RNAseK colocalises with V-ATPase to maintain epithelial membrane structure

To assess RNAseK localisation, we generated an anti-RNAseK antibody. Efficacy was tested by comparing across RNAseK expression levels; the fluorescence intensity of the antibody staining increased upon genetic upregulation (Fig. S14B) and decreased when expression was intentionally genetically reduced (Fig. S14C), compared with control (Fig. S14A), confirming that the detected signals were directly correlated with RNAseK abundance and not due to non-specific binding.

**Fig. 9.**
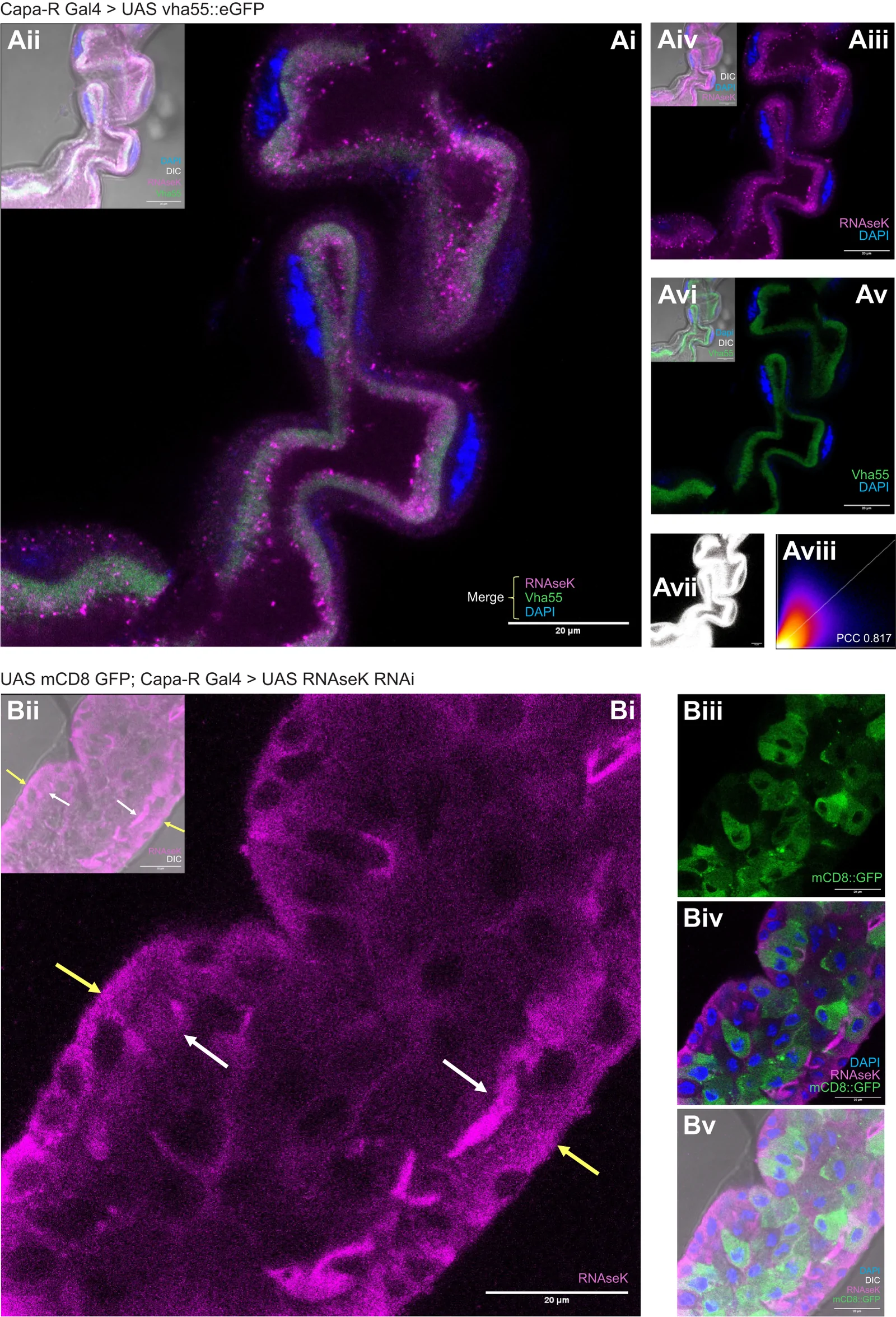
**RNAseK colocalises with apical Vha55 and its depletion causes mislocalisation.** (A) Confocal imaging of control flies: (i) merged staining of RNAseK (magenta), Vha55::eGFP (green) and DAPI (blue); (iii) RNAseK and DAPI only; (v) Vha55 and DAPI only; (vii) regions where RNAseK and Vha55 colocalise; (viii) correspondence between RNAseK and Vha55; (insets ii,iv,vi) the same merge plus differential interference contrast (DIC) to provide structural information. (B) Confocal imaging of RNAseK knockdown flies: (i) staining of RNAseK only; (ii) the same merge with DIC; (iii) staining of mCD8::GFP only; (iv) merged staining of mCD8::GFP, RNAseK and DAPI; (v) merge as for iv with DIC. Yellow arrows indicate basal surfaces; white arrows indicate apical surfaces. Scale bars: 20 μm (Ai–Avii,Bi–Bv); 10 μm (Aviii).

The V-ATPase B-subunit, Vha55, is localised apically in tubule cells and restores fluid transport upon transgenic rescue (Du et al., 2006). Within the tubule, RNAseK exhibited complete apical colocalisation with Vha55 fluorescence (Fig. 9A; Fig. S13A,B), consistent with, though not direct evidence of, direct cooperative interplay (Perreira et al., 2015). Upon RNAseK depletion, RNAseK protein redistributed diffusely from apical to basal membranes (Fig. 9B; Fig. S14D), mirroring polarity-collapse phenotypes reported for V-ATPase loss in mammalian epithelia (Bidaud-Meynard et al., 2022). This suggests the possibility that RNAseK and V-ATPase function as an integrated apical complex essential for epithelial polarity, though does not necessarily exclude other possibilities.

**Fig. 10.**
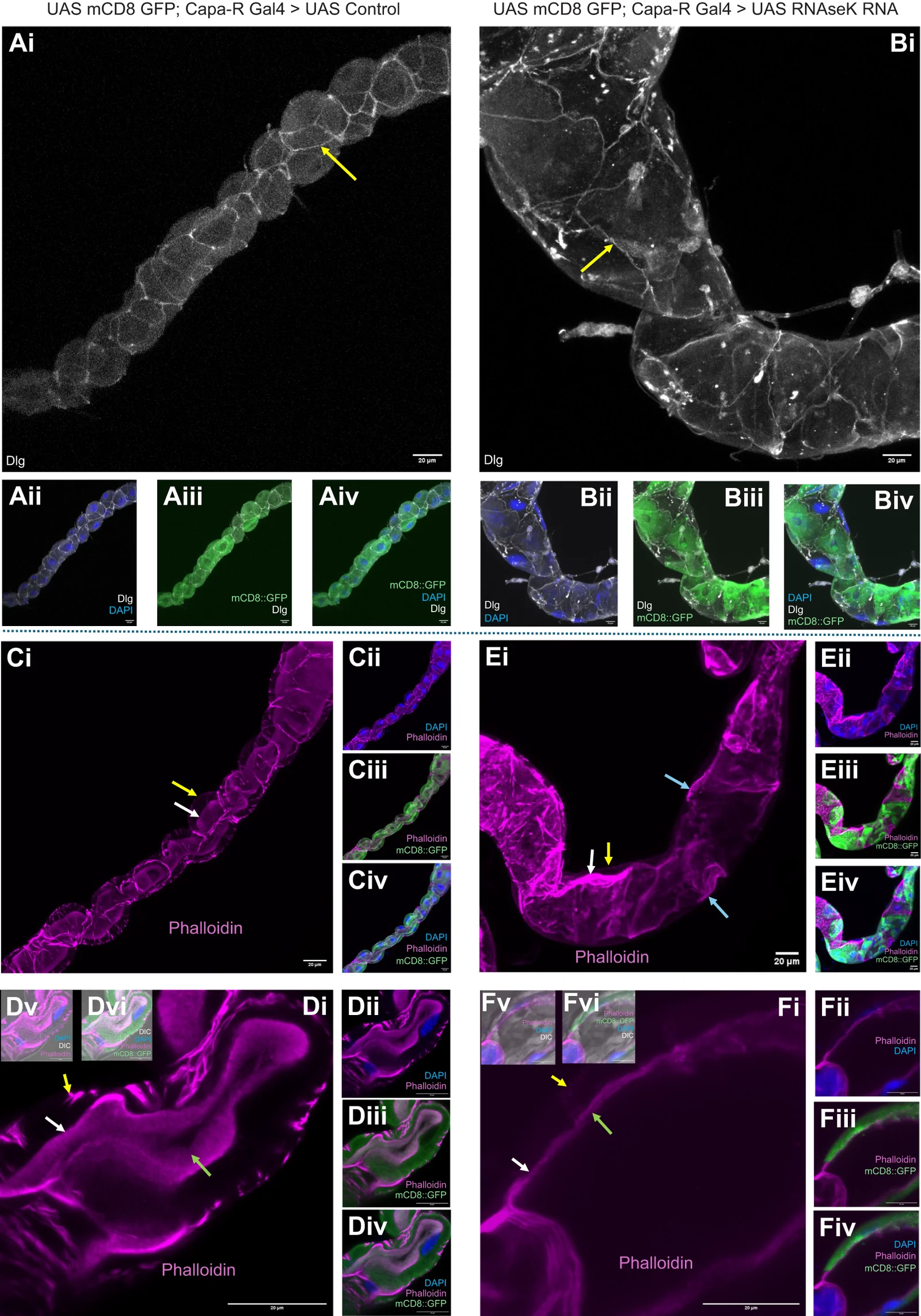
***RNAseK* knockdown disrupts tubule integrity and actin organisation.** (A,B) Confocal investigation of tubule junctional structure in (A) control and (B) RNAseK knockdown flies: (i) staining for Dlg only; (ii) Dlg plus DAPI; (iii) mCD8::GFP plus Dlg; (iv) mCD8::GFP plus Dlg plus DAPI. (C–F) Confocal imaging for actin organisation in Phalloidin-stained tubules from (C,D) Control and (E,F) *RNAseK* knockdown flies, shown at low and high magnification: (i) Phalloidin staining only; (ii) Phalloidin plus DAPI; (iii) Phalloidin plus mCD8::GFP; (iv) Phalloidin plus mCD8::GFP plus DAPI. Scale bars: 20 μm. In all the above cases, inset images show the same merge plus DIC to provide structural information; yellow arrows indicate basal surfaces; white arrows indicate apical surfaces; blue arrows indicate fused apical–basal regions; and green arrows indicate the brush border.

Further, to assess the impact of RNAseK downregulation on cellular arrangement and boundaries, Dlg – a known surface marker within the tubule (Dornan et al., 2023) – was immunostained. Control tubules marked well-defined cell boundaries (Fig. 10A), while RNAseK RNAi tubules displayed irregular cell distribution and arrangement (Fig. 10B). As Dlg interacts with adducin and actin to maintain epithelial architecture and polarisation (Woods et al., 1996; Bilder et al., 2000; Wang et al., 2011), defects upon RNAseK downregulation in the form of disrupted apical F-actin organisation correlated with disrupted Dlg organisation (Fig. 10E; Fig. S12D). *RNAseK* knockdown affects epithelial integrity and leads to fused apical–basal layers observed under magnified views (Fig. 10E, blue arrow); it also affects brush-border integrity (Fig. 10F; Fig. S12D). This disintegration confirms global epithelial disarray upon RNAseK depletion, potentially linking vesicular acidification failure to polarity complex instability.

## DISCUSSION

RNAseK has recently emerged as a V-ATPase-associated component in mammalian systems, with structural analyses placing it in the V0 sector and functional work linking it to lysosomal hydrolase delivery and autophagosome degradation (Perreira et al., 2015; Abbas et al., 2020; Makar et al., 2024). Here, we have provided computational and biomolecular evidence that this role for RNAseK is broadly conserved across evolutionary history, and RNAseK remains a crucial epithelial determinant in the fruit fly *Drosophila melanogaster* (Allan et al., 2005; Mo et al., 2020).

At the sequence level, the functional elements of RNAseK are strongly conserved even in species as distant from the fly as humans and zebrafish, suggesting that these regions serve vital biological functions. One may hypothesise that this function is linked to the recent reports of RNAseK as a regulatory component of the V-ATPase complex (Perreira et al., 2015; Makar et al., 2024), especially given that we have located multiple dCLEAR cis-regulatory elements in close proximity with the *RNAseK* gene, suggesting a shared transcriptional programme with the epithelial *vha* genes (Bouché et al., 2016).

This nuance is important for interpreting the *Drosophila* data: although mammalian studies support a close relationship between RNAseK and the V-ATPase, they also indicate that RNAseK-dependent phenotypes may reflect altered V-ATPase-associated trafficking, organelle maturation or membrane homeostasis rather than a simple, global loss of proton-pump activity. We therefore interpret the overlapping *Drosophila* RNAseK and V-ATPase phenotypes as evidence for disruption of an acidification-dependent epithelial pathway, not as direct biochemical proof of reduced V-ATPase activity.

Our molecular experimentation supports the hypothesis that RNAseK is crucial in the major epithelial tissues within the fruit fly – namely, the midgut, hindgut, Malpighian tubules and salivary glands – in a fashion consistent with impaired acidification and acidification-dependent membrane trafficking, processes classically driven by V-ATPase activity. Indeed, the major biological disruptions caused by RNAseK manipulation can broadly be linked with those described in V-ATPase disruption, including loss of polarisation during embryogenesis, suggesting RNAseK may support epithelial morphogenesis as well as adult maintenance (Davies et al., 1996; Wieczorek et al., 2009), disruption of gut polarity and acidification (Overend et al., 2016).

Indeed, our observation that bafilomycin treatment collapses the alkaline midgut domain aligns with models where V-ATPase energises both acidic and alkaline regions through secondary transport mechanisms (Boudko et al., 2001). This is further supported by reduced starvation resistance (Cannell et al., 2016), disruption of tubule transport systems (Cabrero et al., 2020), impaired tubular fluid secretion and hormone response (Davies et al., 2013), and defective secretory granule maturation in salivary glands (Farkaš et al., 2015), consistent with the observed feeding defect and defective V-ATPase membrane localisation (Bidaud-Meynard et al., 2022). Importantly, the phenotypic similarity between *RNAseK* knockdown and V-ATPase inhibition should be interpreted as convergence on an acidification-dependent epithelial pathway, rather than proof that the two perturbations produce identical primary lesions (Perreira et al., 2015; Overend et al., 2016; Wang et al., 2021).

The exquisite dosage sensitivity of RNAseK can be seen in the fact that both reduction and elevation of its levels in principal cells abolish normal xanthine pigmentation of luminal concretions. This balance may reflect not only V-ATPase support but also potential RNAseK-mediated regulation of pigment vesicles and epithelial insertion.

That said, only depletion elicits crystal precipitation and induction of calcium-responsive genes such as *SPoCk* and *Crystallin* (Southall et al., 2006; Chung and Turney, 2017). These mineralisation-associated changes probably arise downstream of RNAseK-dependent disruption of V-ATPase-linked luminal acidification and solute handling, although altered calcium homeostatic or vesicular trafficking pathways may also contribute (Southall et al., 2006; Chung and Turney, 2017). This transcript-sensitive response suggests that a specific stoichiometric balance of RNAseK is required to maintain the vesicular or luminal pH range for orderly urate deposition; any deviation disrupts pigment incorporation, while excess RNAseK apparently maintains sufficient acidification to prevent uncontrolled nucleation.

In stellate cells, reduced cell number, distorted morphology and reduced basal and CAPA-stimulated fluid secretion further suggest that RNAseK may support V-ATPase linked trafficking or membrane homeostasis required for epithelial cell integration and tubule maintenance (Cabrero et al., 2014, 2020; Perreira et al., 2015). A more direct effect on ClC-a or aquaporin abundance/localisation also remains possible and could contribute to the impaired secretion phenotype in parallel with V-ATPase-linked membrane homeostatic defects.

At the organismal level, the divergent starvation and desiccation responses, together with abdominal water retention following principal and stellate cell RNAseK manipulation, suggest that RNAseK depletion produces integrated, cell type-specific disruption of epithelial transport and fluid balance rather than a purely cell-autonomous or uniform tubule-wide failure (Cannell et al., 2016; Cabrero et al., 2020).

The Smurf phenotype and dye leakage observed upon RNAseK knockdown provide functional evidence consistent with a breach in epithelial barrier integrity. While Smurf positivity can represent the net outcome of a leak/transport cycle involving both paracellular influx and active dye clearance by transport epithelia (Livingston et al., 2020), the concurrent cytoskeletal collapse observed here supports barrier failure, similar to the chronic epithelial leakage seen in aged flies and septate junction mutants. This phenomenon may represent a secondary consequence of V-ATPase destabilisation, which can disrupt the endocytic trafficking of junctional components required for membrane integrity (Rera et al., 2012; Salazar et al., 2023; Dornan et al., 2023; Allan et al., 2005; Petzoldt et al., 2013), although a more direct role for RNAseK in junctional integrity remains possible. These findings suggest that RNAseK is required for adult epithelial barrier maintenance, whether through V-ATPase-associated membrane homeostasis, direct effects on junctional pathways, or both.

Likewise, the cortical F-actin collapse seen in the midgut upon RNAseK depletion mirrors phenotypes produced by disruption of the WASH–Arp2/3 actin-nucleation pathway (Carnell et al., 2011), suggesting that RNAseK supports V-ATPase-dependent retrieval or recycling of actin-regulatory complexes. Alternatively, RNAseK could influence actin organisation more directly (e.g. by affecting actin regulatory transcripts or membrane-cytoskeletal coupling), with V-ATPase mislocalisation arising secondarily from polarity collapse. Together, these data indicate that RNAseK is required for steady-state cytoskeletal homeostasis in epithelia, while leaving open whether this is mediated primarily through V-ATPase-dependent trafficking, direct regulation of actin-associated pathways or secondary effects of polarity collapse. Hindgut-specific RNAseK downregulation causes localised swelling immediately anterior to the tubule–ureter junction, accompanied by fluid backflow, retention and white/black deposits just posterior to the ureter junction that occlude the lumen. This is explained by the potential loss of the strong electrogenic H^+^ gradient normally generated by hindgut V-ATPase, which abolishes the driving force for K^+^/H^+^ exchange and Cl^−^-driven water reabsorption, resulting in osmotic water retention and precipitation of undissolved waste (Shanbhag and Tripathi, 2005; Overend et al., 2016).

Finally, the ability of RNAseK depletion to trigger rapid basolateral mislocalisation of Vha55 and brush-border dissolution (Du et al., 2006; Dornan et al., 2023) identifies RNAseK as the possible apical tether or trafficking support factor for the holoenzyme in post-mitotic epithelia. As such, while we have not directly proven molecular interaction, we have gathered a bevy of correlative genetic, phenotypic, functional and localisation evidence supporting the notion that RNAseK is absolutely required in epithelial tissues. One hypothesis, supported by orthology, phenotypic similarities and co-localisation, is that RNAseK supports the normal functioning of the V-ATPase holoenzyme. That said, we cannot definitively confirm this to be the case, and other possibilities, such as direct catalytic action of RNAseK within cells, cannot be excluded. Regardless, the finding that RNAseK is such a critical epithelial effector is not only highly relevant in *Drosophila* but, given the high degree of functional RNAseK homology across evolutionary history, also has implications for mammalian physiology and medical science.

## Supplementary Material

10.1242/jexbio.252529_sup1 Supplementary information
