# Supplementary material for: RNAseK is vital for epithelial function in Drosophila melanogaster
Source: J Exp Biol. 2026 Sep 3;229(17):jeb252529. doi: 10.1242/jeb.252529 (PMC13615687; doi:10.1242/jeb.252529)
Supplement: Supplementary file 1 [file jexbio-229-252529-s1.pdf]

A

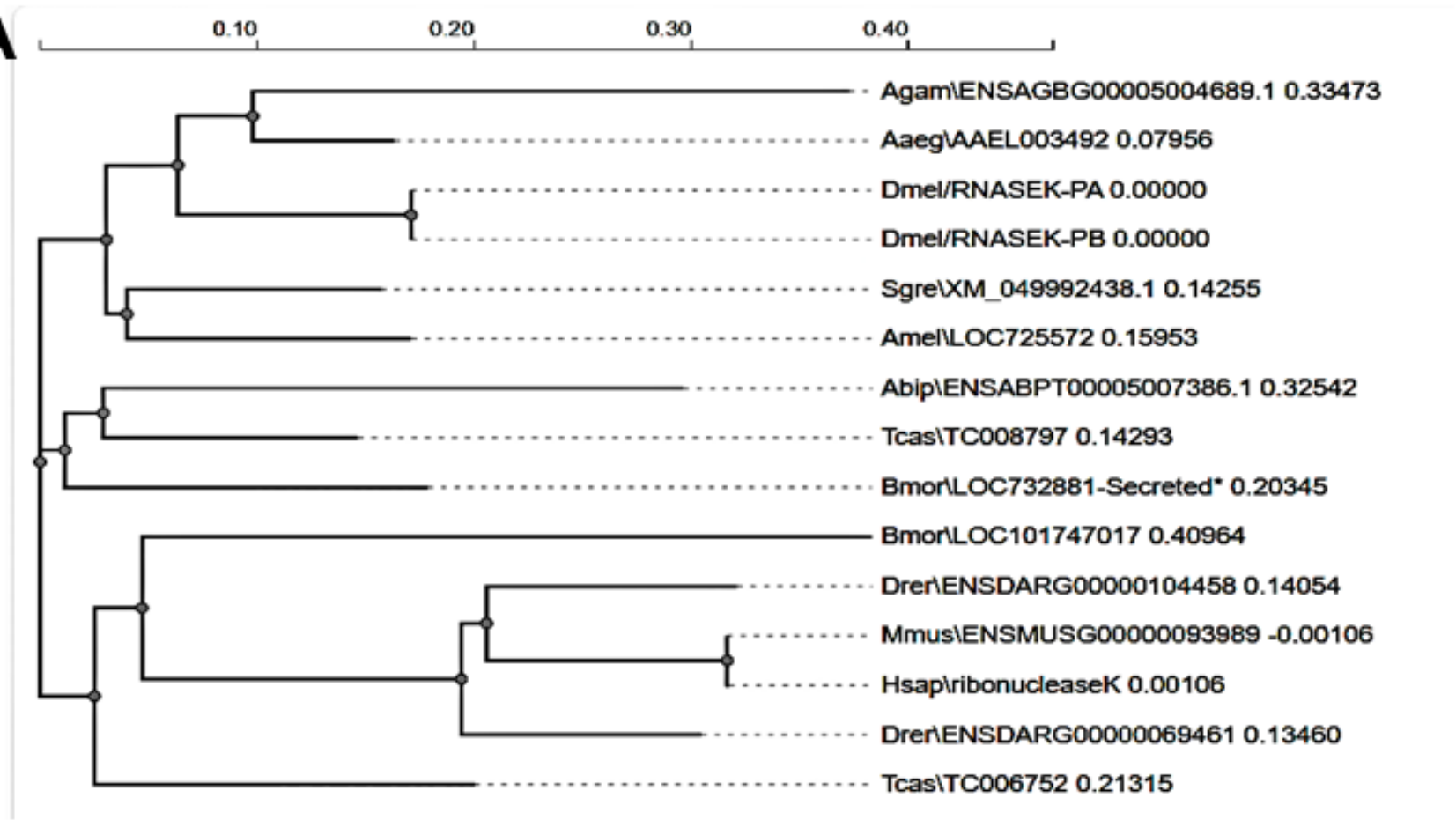

B

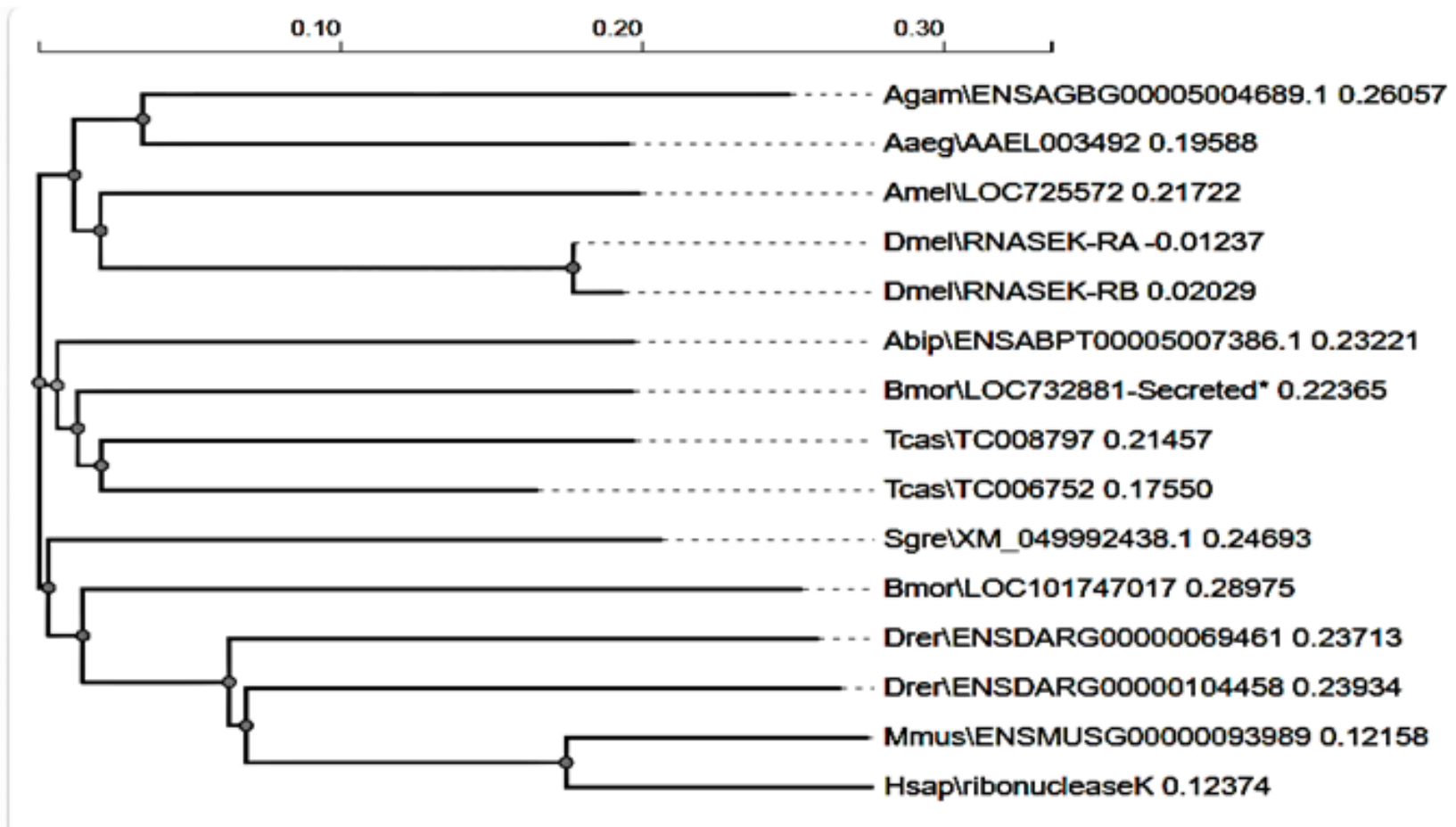

C

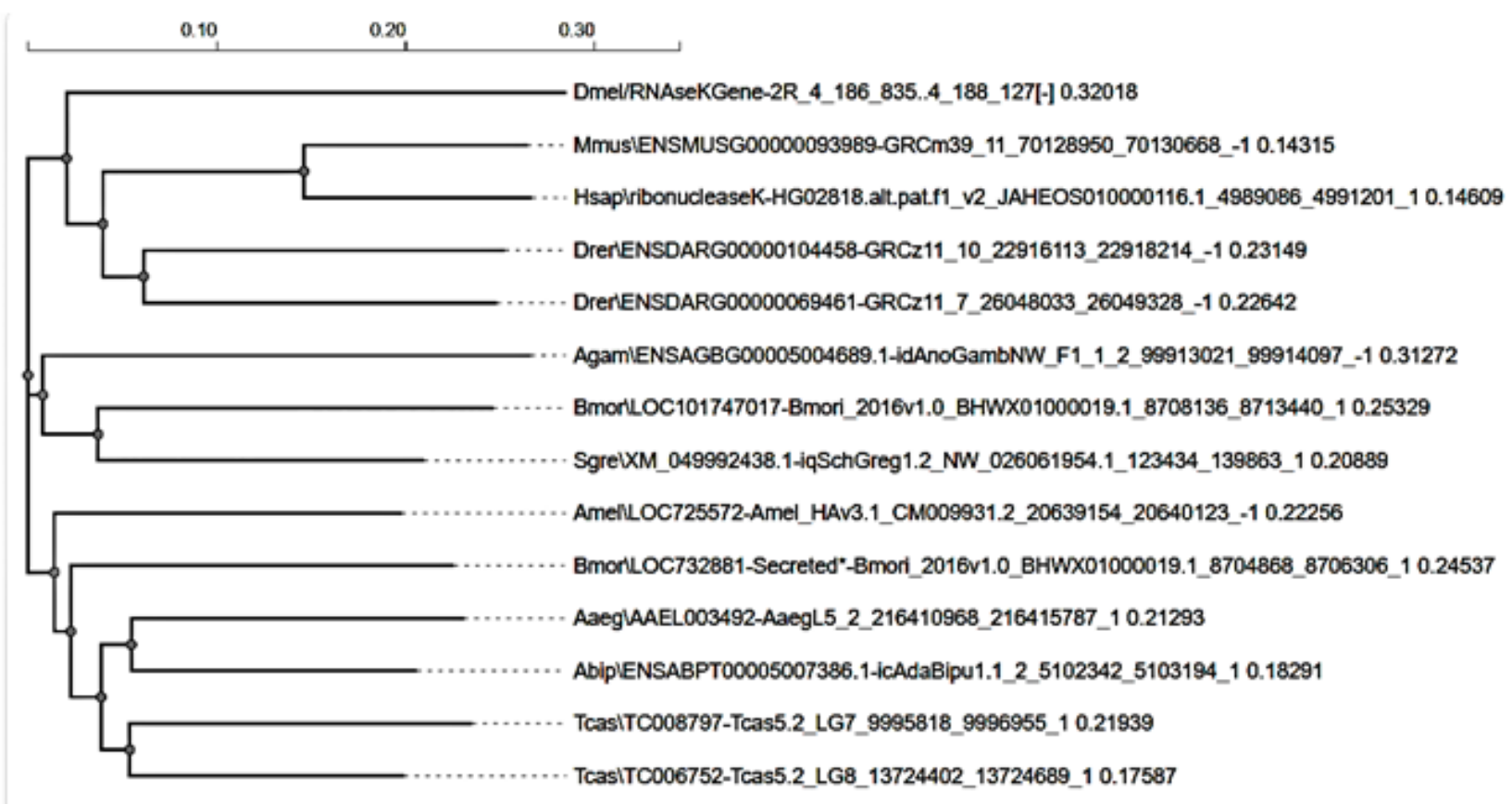

Journal of Experimental Biology • Supplementary information

Fig. S1. Divergence of RNAseK sequences.

Phylogenetic trees contructed based on conservation of the RNAseK A – protein; B – cDNA and C – gene sequences across selected other species. Multiple Sequence Alignments and tree construction were carried out using Muscle5 <ref no>. In all cases, sequences are shown in the format species\sequence name.

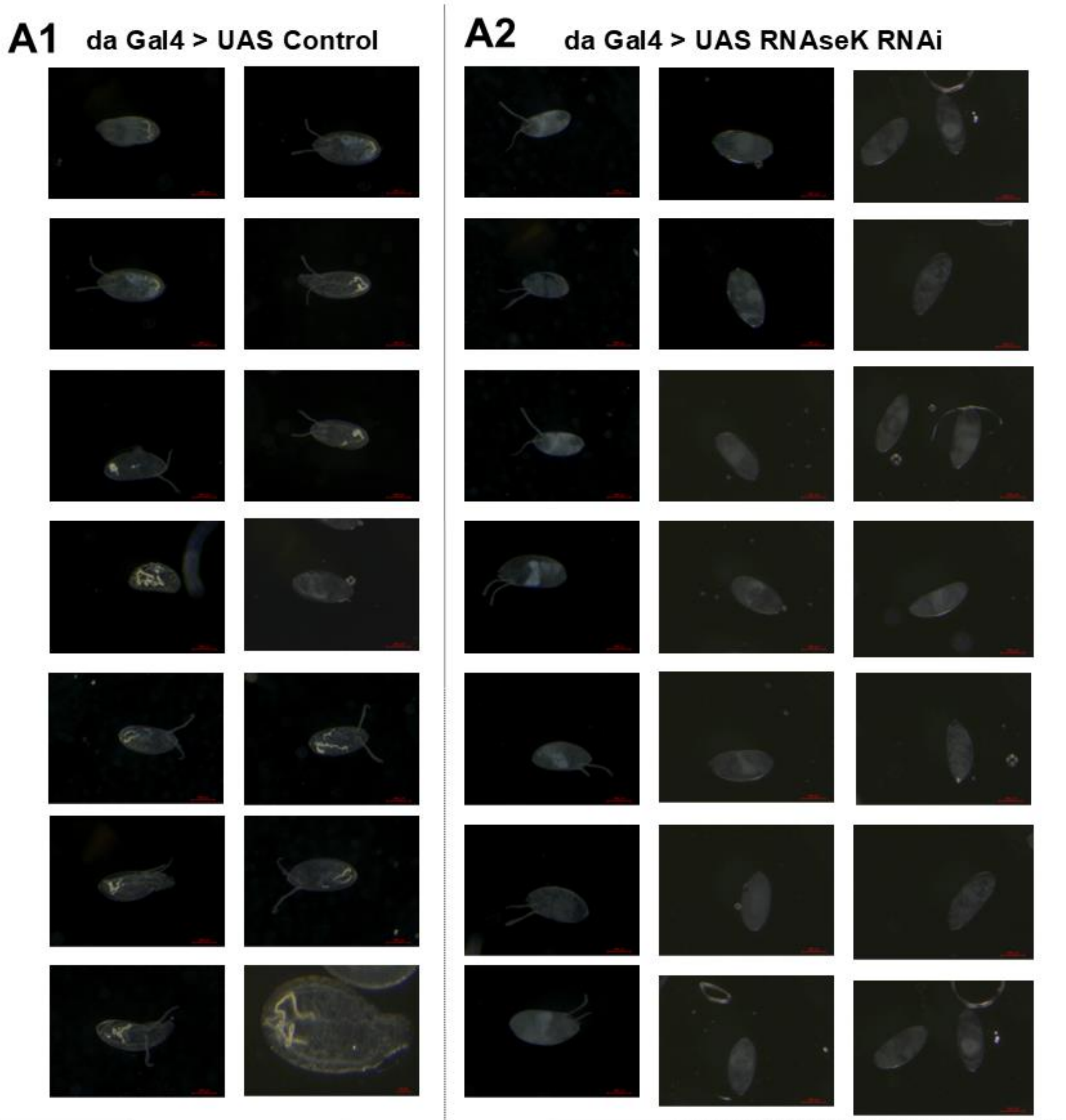

B P. Cross : da Gal4 > UAS RNAseK RNAi / TM3,Sb (BL 35779)

Replicate	Total Eggs Laid	Eggs Hatched	% Hatching	Eggs Not Hatched	% Not Hatched
Replicate 1	110	57	51.8%	53	48.2%
Replicate 2	96	47	48.9%	49	51.1%
Replicate 3	52	26	50.0%	26	50.0%
Replicate 4	122	64	52.5%	58	47.5%

↑ da Gal4 > TM3,Sb ↑ da Gal4 > UAS RNAseK RNAi

Journal of Experimental Biology • Supplementary information

Fig. S2. Ubiquitous RNAseK depletion causes embryonic lethality and failure of Malpighian tubule formation

(A1), 20–22 h old experimental control embryo showing normal development and proper formation of Malpighian tubules and other internal organs. (A2), 20–22 h old embryo expressing RNAseK RNAi under a ubiquitous Gal4 driver (da Gal4) exhibiting complete loss of internal organ formation, including the absence of Malpighian tubules. (B), quantification table showing embryonic lethality in RNAseK RNAi embryos driven by da Gal4; approximately 50% of total embryos hatched, and all surviving adults displayed the TM3,Sb phenotype, confirming the absence of homozygous transgenics. Scale bars: 200µm.

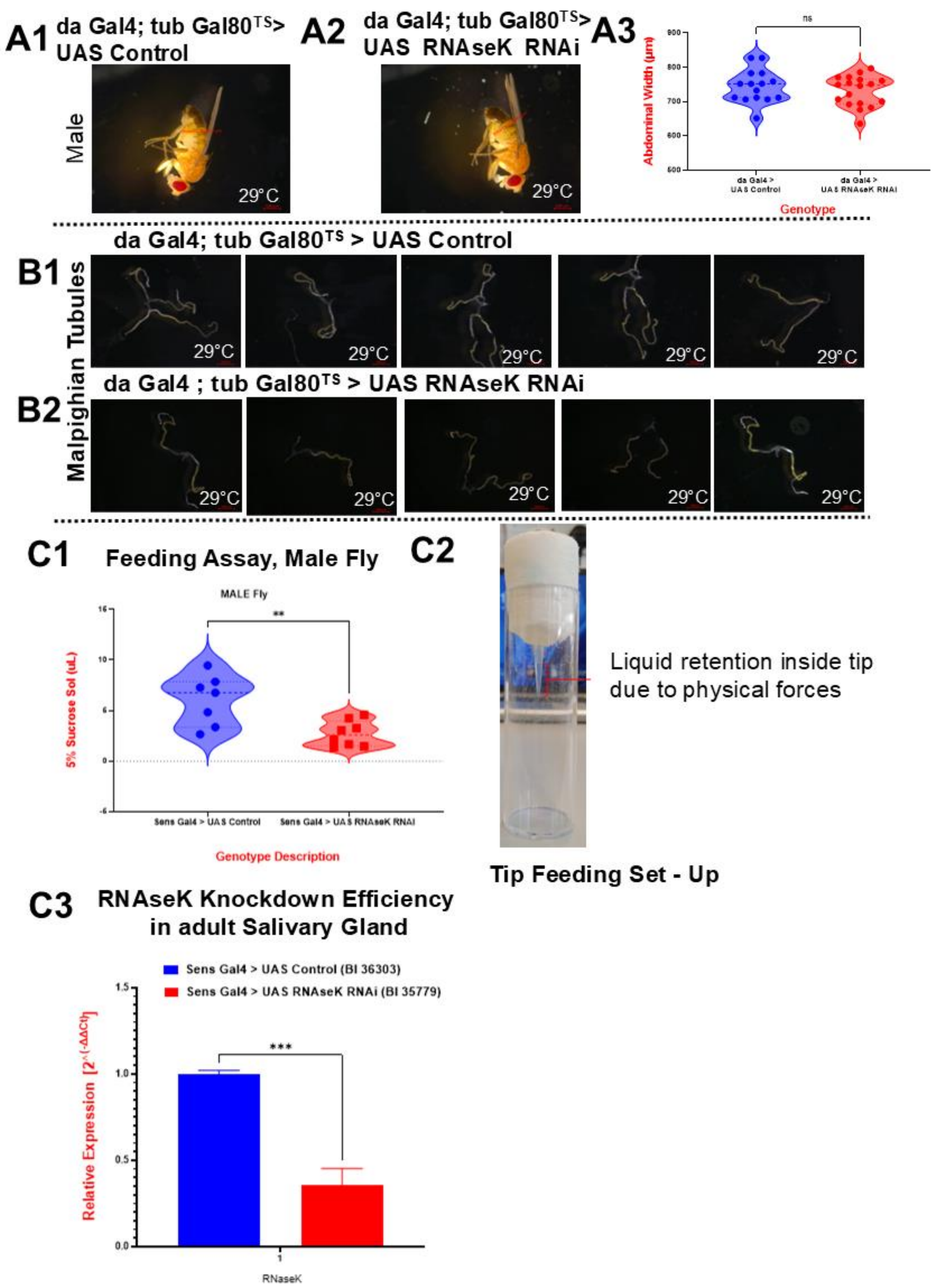

Journal of Experimental Biology • Supplementary information

Fig. S3. Adult RNAseK depletion preserves gross tubule morphology but constitutive salivary gland-specific knockdown impairs feeding.

(A1) and (A2), adult male flies reared at 18 °C until eclosion and transferred to 29 °C for 9-days before imaging. (A1), control genotype; (A2), ubiquitous RNAseK knockdown. (A3), graph comparing abdominal width between control and RNAseK RNAi male flies showing no significant difference (MannWhitney test). (B1), control adult Malpighian tubule showing normal morphology when reared at 29 °C. (B2), Malpighian tubule from flies with ubiquitous RNAseK knockdown showing no gross morphological defects compared to controls at 29 °C. (C1), feeding assay comparing control and salivary gland specific RNAseK knockdown in 9-days old males. RNAseK knockdown significantly reduces feeding efficiency ($p \leq 0.01$, MannWhitney test). (C2), A schematic of the feeding assay setup using a 200 µL tip containing an internal filter, which holds liquid due to physical forces, was used to feed flies. (details in Methods). (C3), qPCR showing significant RNAseK downregulation in the salivary gland tissue (*, $p \leq 0.001$), consistent with the observed feeding defect. Scale bars: 500µm.

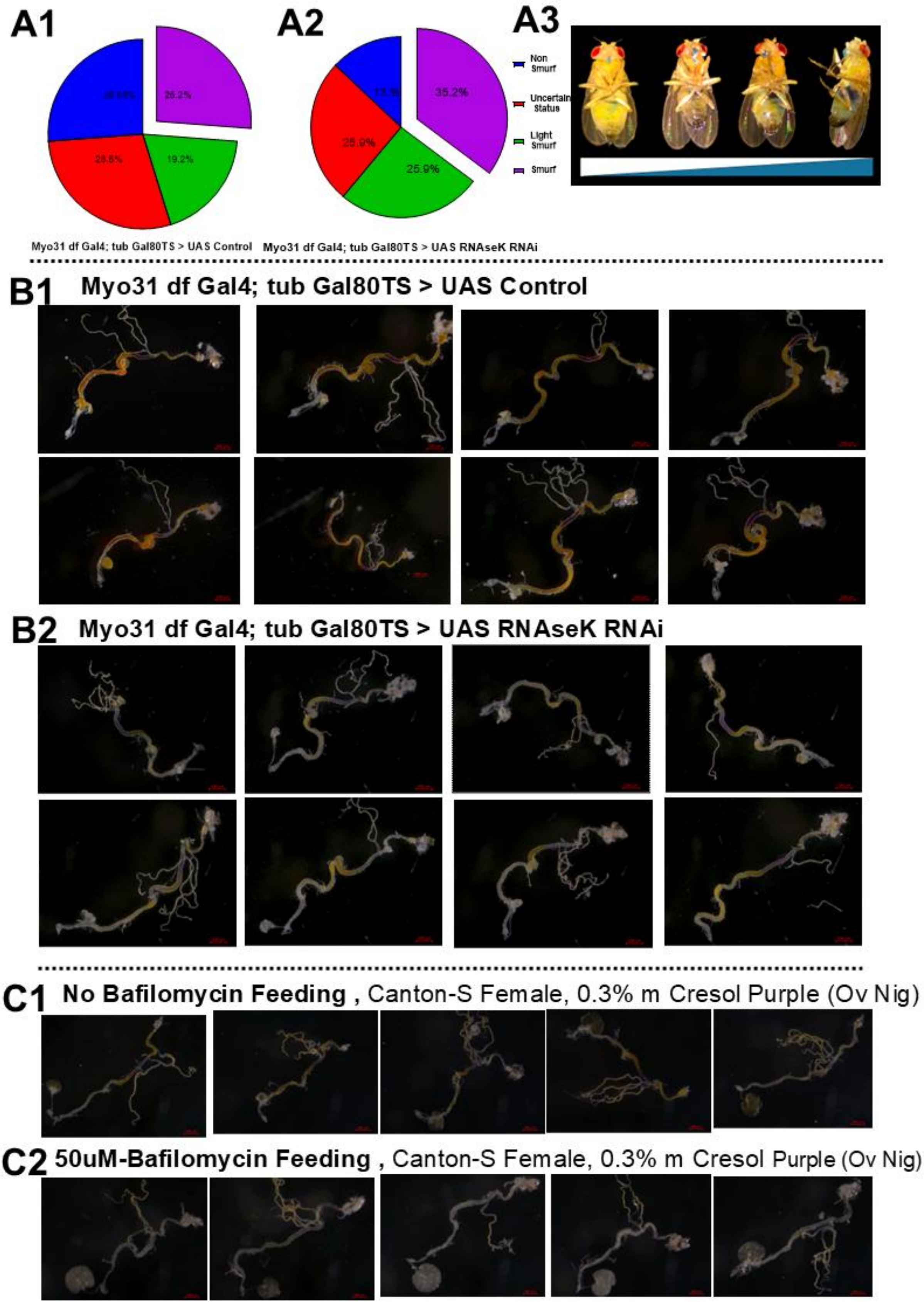
A1
26.2%
28.8%
19.2%
A2
35.2%
25.9%
25.9%
A3
Non Smurf
Uncertain Status
Light Smurf
Smurf
Myo31 df Gal4; tub Gal80TS > UAS Control
Myo31 df Gal4; tub Gal80TS > UAS RNAseK RNAi
B1 Myo31 df Gal4; tub Gal80TS > UAS Control
B2 Myo31 df Gal4; tub Gal80TS > UAS RNAseK RNAi
C1 No Bafilomycin Feeding , Canton-S Female, 0.3% m Cresol Purple (Ov Nig)
C2 50uM-Bafilomycin Feeding , Canton-S Female, 0.3% m Cresol Purple (Ov Nig)

Fig. S4. Gut-specific RNAseK depletion induces Smurf phenotypes and disrupts gut pH partitioning.

(A1), pie chart showing the proportion of Smurf and healthy flies in the control group after 9-days at 29 °C. (A2), equivalent quantification for gut-specific RNaseK RNAi flies. (A3), representative fly images used to score Smurf classification into four groups: Non Smurf, Uncertain, Light Smurf, and Smurf. (B1), pH partitioning in control females (9-days at 29 °C after eclosion, 18 °C before eclosion) . (B2), pH partitioning in gut specific RNAseK RNAi females (9-days at 29 °C after eclosion, 18 °C before eclosion), showing marked alteration. (C1), pH partitioning of Canton-S females maintained on 5% sugar diet and fed 0.3% mcresol purple overnight. (C2), Canton-S females fed 5% sugar + 50 µM bafilomycin for one week and 0.3% mcresol purple overnight, exhibiting a disturbed pH pattern. Scale bars: 500µm.

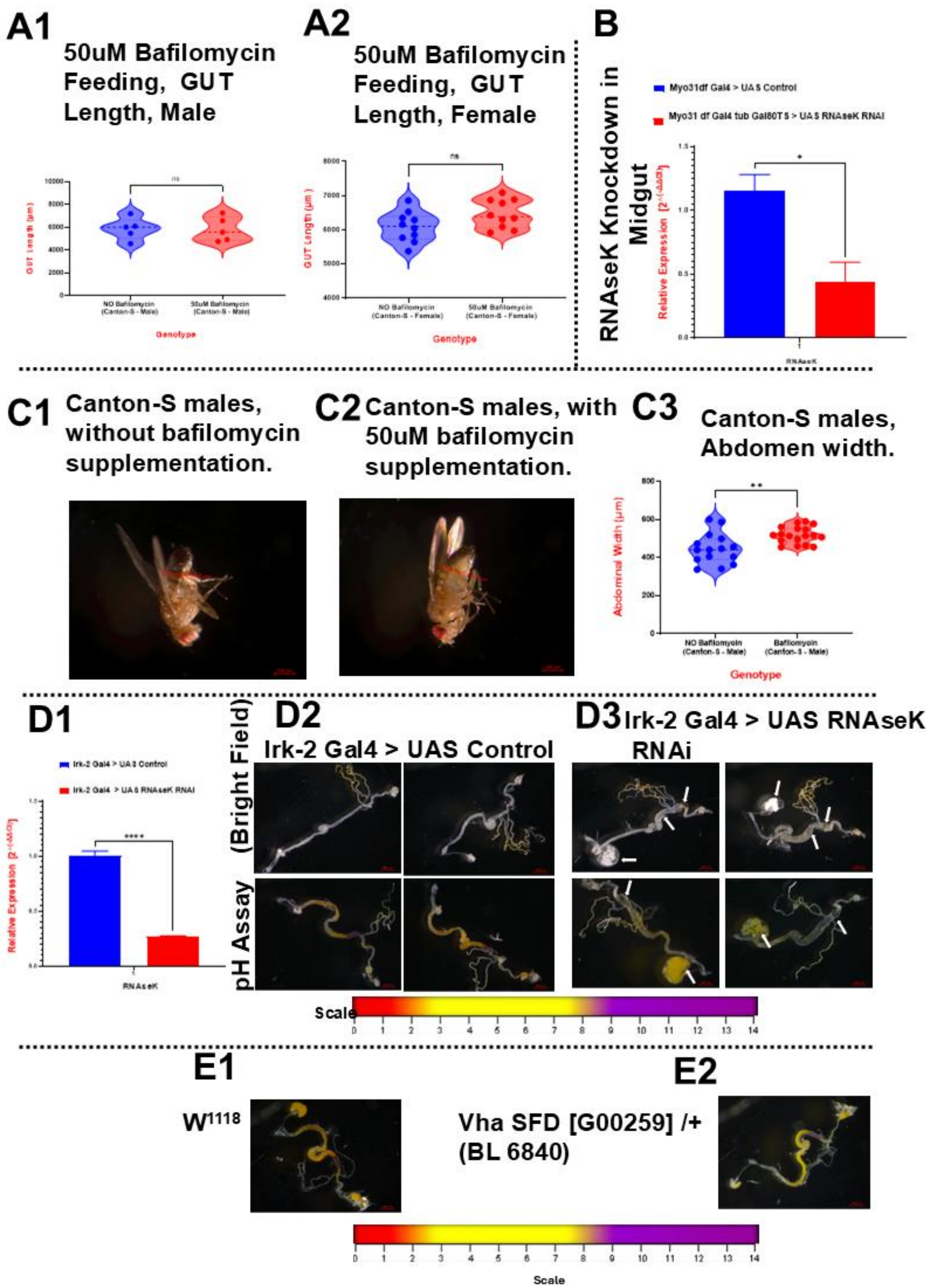

Journal of Experimental Biology • Supplementary information

Fig. S5. RNAseK depletion and V-ATPase impairment disrupt gut pH partitioning, gut morphology and abdominal homeostasis.

(A1), gut length in adult males and (A2) in females fed 50 µM bafilomycin diet, showing no significant differences (MannWhitney test). (B), relative RNAseK expression under Myo31df Gal4 (mid-gut driver) confirming knockdown (*, $p \leq 0.05$). (C1), Canton-S male controls fed 5% sugar; (C2), males fed 5% sugar + 50 µM bafilomycin; (C3), graph showing a significant increase in abdominal width in bafilomycin fed males (**, $p \leq 0.01$, MannWhitney). (D1), RNAseK expression significantly reduced in hindgut (Irk2 Gal4 driver, ****, $p \leq 0.0001$). (D2–D3), bright field and pH partitioning images showing severe pH disruption and swelling of midgut and hindgut (white arrows), including crop enlargement upon RNAseK knockdown. (E1), normal pH gradient in w1118 controls; (E2), disrupted gradient and loss of acidic red midgut zone in VhaSFD mutants, correlated with impaired V-ATPase function (Overend et al., 2016). Scale bars: 500µm.

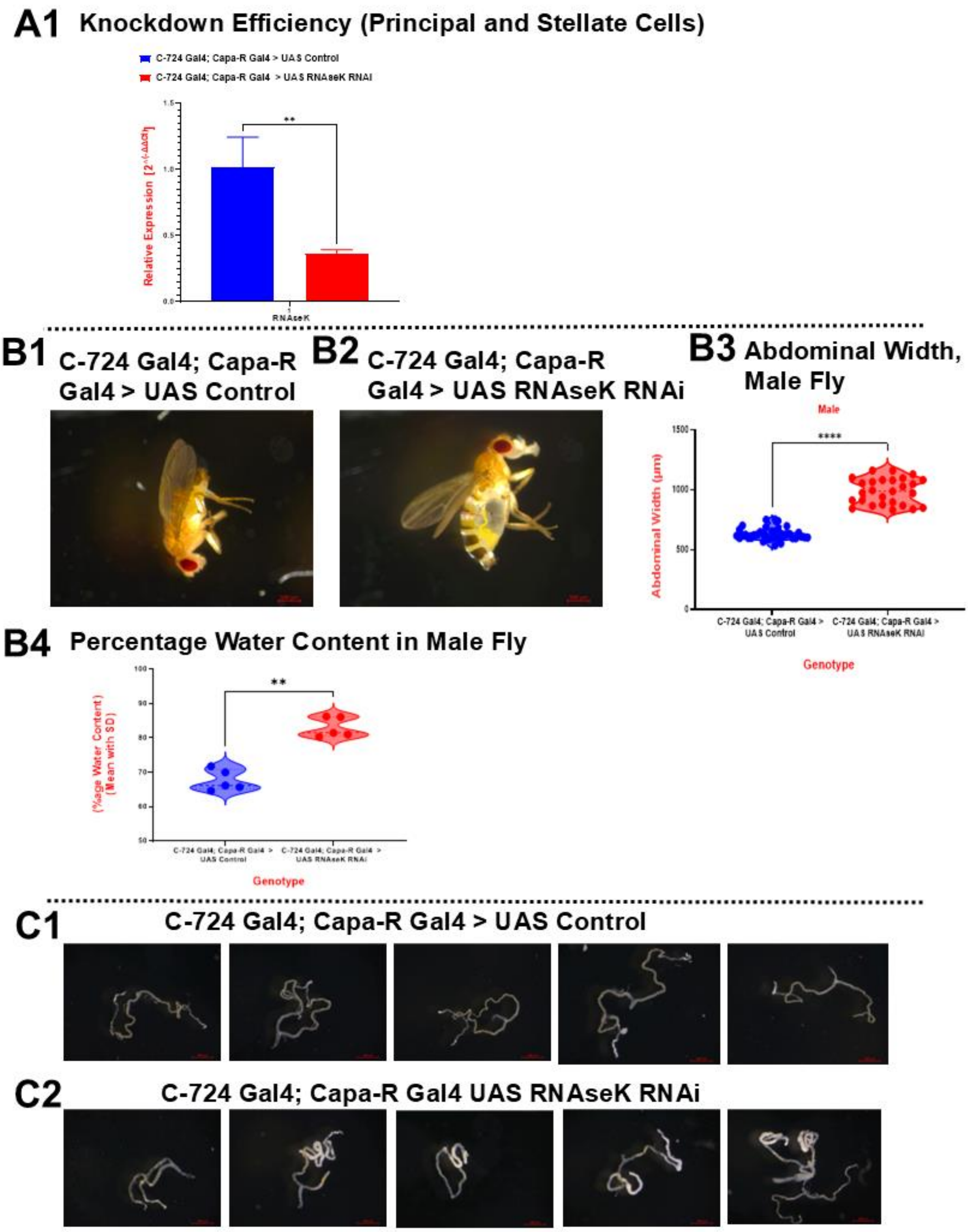
A1
Knockdown Efficiency (Principal and Stellate Cells)
C-724 Gal4; Capa-R Gal4 > UAS Control
C-724 Gal4; Capa-R Gal4 > UAS RNAseK RNAi
Relative Expression
RNAseK
B1
C-724 Gal4; Capa-R Gal4 > UAS Control
B2
C-724 Gal4; Capa-R Gal4 > UAS RNAseK RNAi
B3
Abdominal Width, Male Fly
Male
Abdominal Width (µm)
C-724 Gal4; Capa-R Gal4 > UAS Control
C-724 Gal4; Capa-R Gal4 > UAS RNAseK RNAi
Genotype
B4
Percentage Water Content in Male Fly
(%age Water Content) (Mean with SD)
C-724 Gal4; Capa-R Gal4 > UAS Control
C-724 Gal4; Capa-R Gal4 > UAS RNAseK RNAi
Genotype
C1
C-724 Gal4; Capa-R Gal4 > UAS Control
C2
C-724 Gal4; Capa-R Gal4 UAS RNAseK RNAi

Fig. S6. Combined principal- and stellate-cell RNAseK knockdown causes abdominal swelling, water retention and cystic Malpighian tubule defects.

(A1) shows the relative expression of RNAseK upon genetic knockdown in principal and Stellate Cells both, confirming significant downregulation (**, $p \leq 0.01$, MannWhitney test). (B1) presents the image of a control male fly, while (B2) shows a 9-days old male with combined RNAseK RNAi in both cell types, displaying a visibly swollen water retained abdomen. (B3) quantifies this phenotype, revealing a significant increase in abdominal width (****, $p \leq 0.0001$), and (B4) compares the percentage water content, showing higher values under RNAseK knockdown conditions (**, $p \leq 0.01$). (C1) depicts a normal control tubule, whereas (C2) shows a tubule with RNAseK downregulation in both principal and stellate cells, exhibiting cyst like structures and white deposits within the lumen and loss of pigment. Scale bars: 500µm.

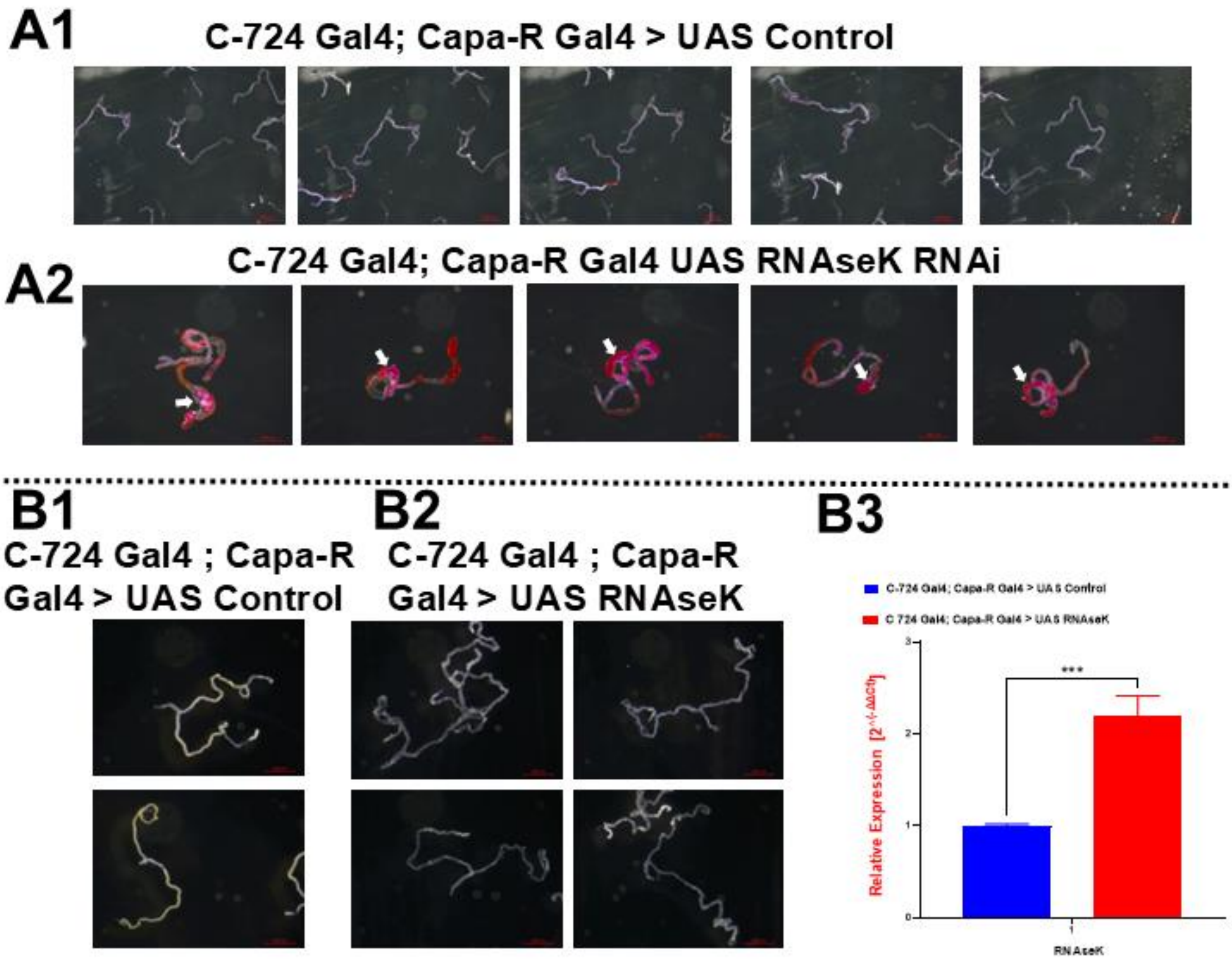

Fig. S7. RNAseK depletion and overexpression differentially disrupt tubule crystal deposition and pigmentation.

(A1 and A2) display Alizarin red stained tubules from control and RNAseK RNAi flies, respectively; control tubules show little or no crystal deposition, while knockdown tubules display distinct red stained crystalline accumulations (marked with white arrows). (B1) shows normal tubules from the control group, and (B2) shows polarised light images of tubules overexpressing RNAseK in both cell types, which appear colourless and lack normal pigmentation. (B3) confirms a significant increase in RNAseK expression upon overexpression (***, $p \leq 0.001$). Scale bars: 500µm.

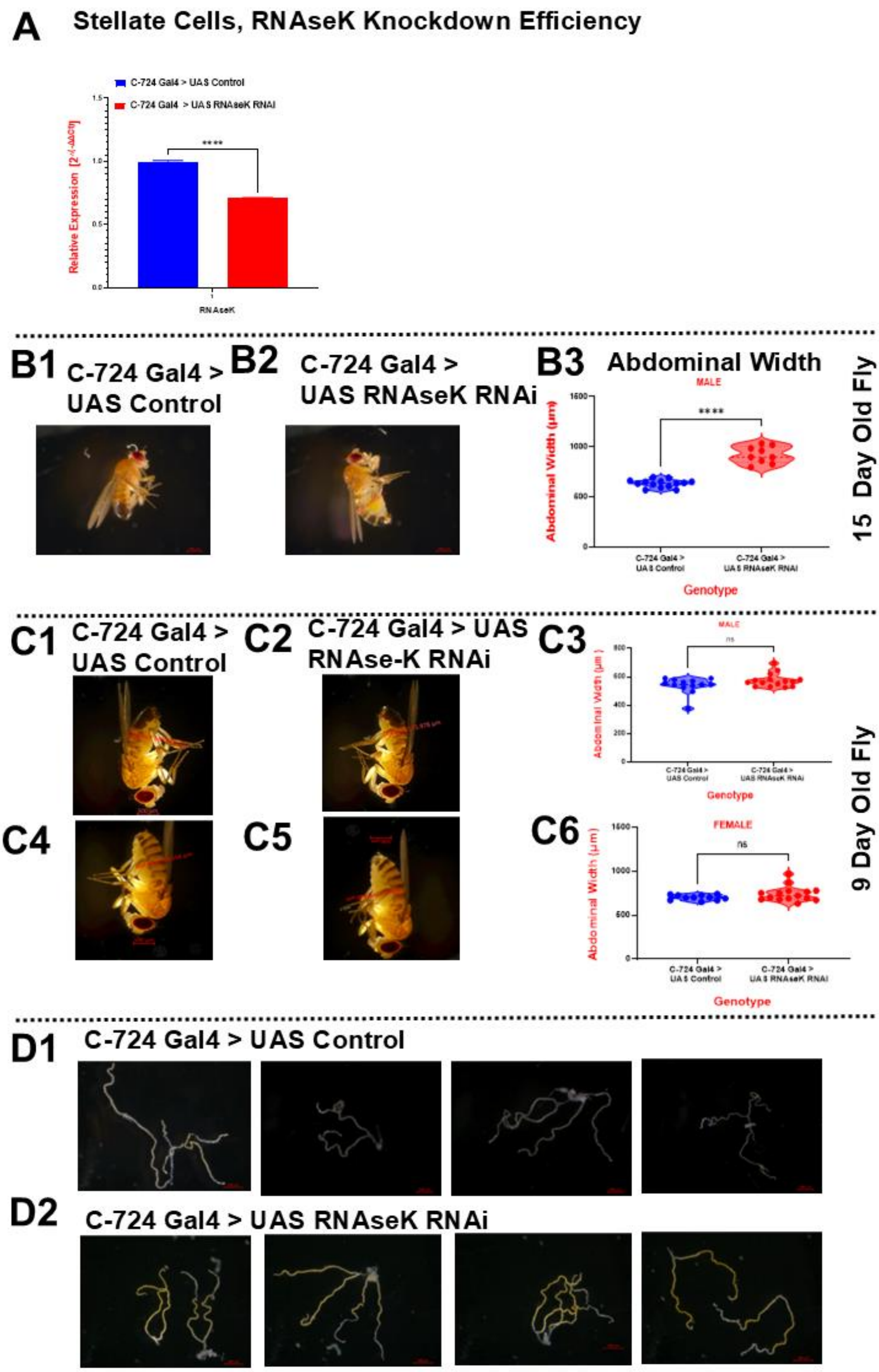

Journal of Experimental Biology • Supplementary information

Fig. S8. Stellate-cell-specific RNAseK knockdown causes delayed abdominal swelling without cystic tubule morphology.

(A) shows the relative expression levels of RNAseK upon stellate cell specific RNAi, confirming significant downregulation (****, $p \leq 0.0001$, Mann–Whitney test). (B1) and (B2) depict 15-days old control and stellate cell specific RNAseK RNAi male flies, respectively, while (B3) shows the corresponding graph revealing a significant increase in abdominal width in RNAseK RNAi males (****, $p \leq 0.0001$). (C1–C6) compare male and female flies at 9-days of age, where no significant difference in abdominal width is observed upon Stellate cell specific knockdown between controls and experimental groups. (D1) and (D2) show representative tubule images from control and stellate cell specific RNAi flies, both exhibiting normal morphology and pigmentation. Scale bars: 500µm

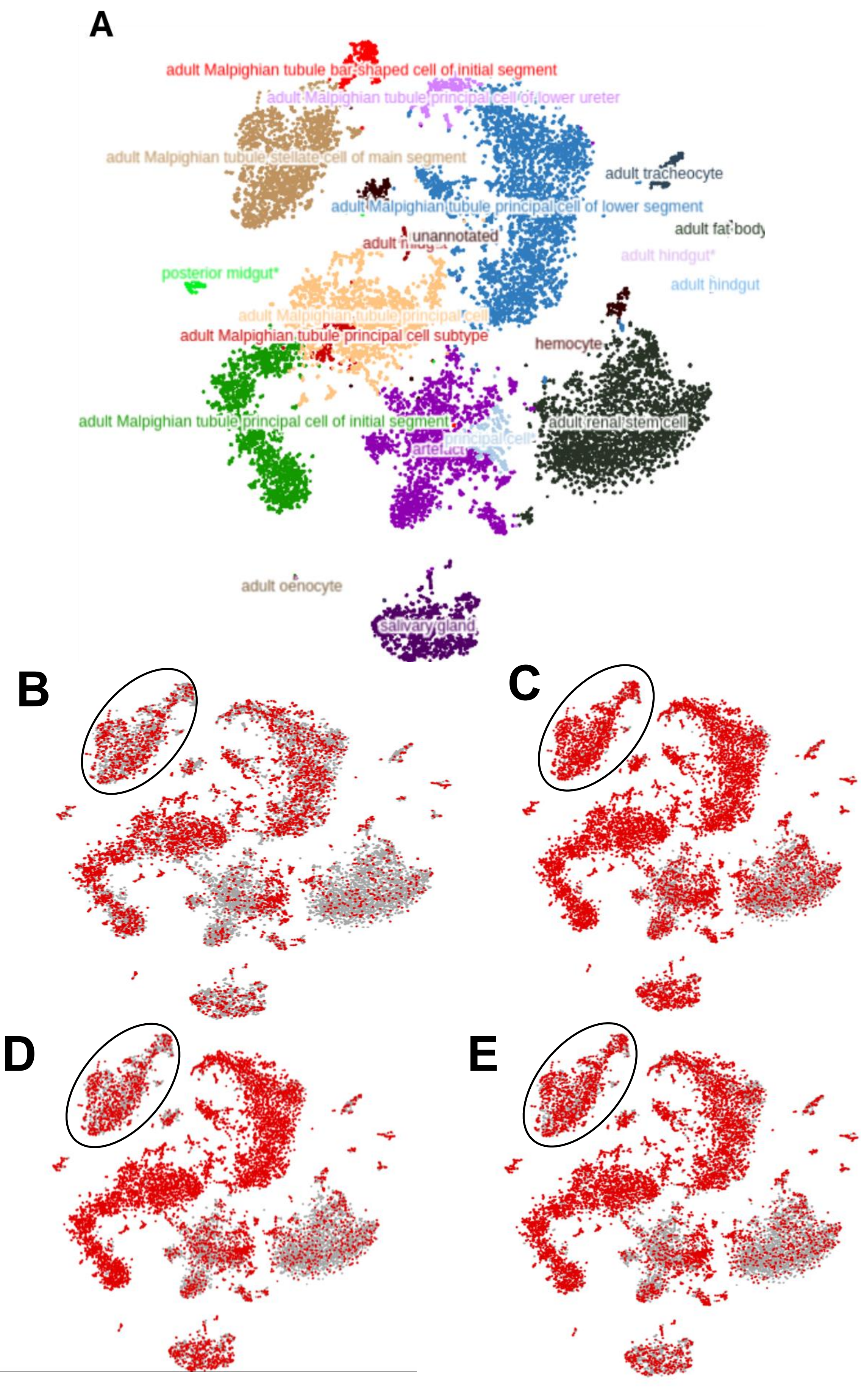

Journal of Experimental Biology • Supplementary information

Fig. S9. RNAseK and V-ATPase subunits are expressed throughout the Malpighian tubule. t-SNE plots of single cell RNA sequencing data taken from the Malpighian tubule samples from the FlyCellAtlas project. A) Cell type annotation of each cluster, for reference. B-E) Cells coloured based on expression of B) RNAseK; C) Vha55; D) Vha100-2; E) VhaAC45. In all cases, this has been reduced to a binary – cells are coloured red if any transcript has been detected at all, and grey if no transcript is present. Figures were generated using the SCope data viewer (https://scope.aertslab.org/#/FlyCellAtlas/*/).

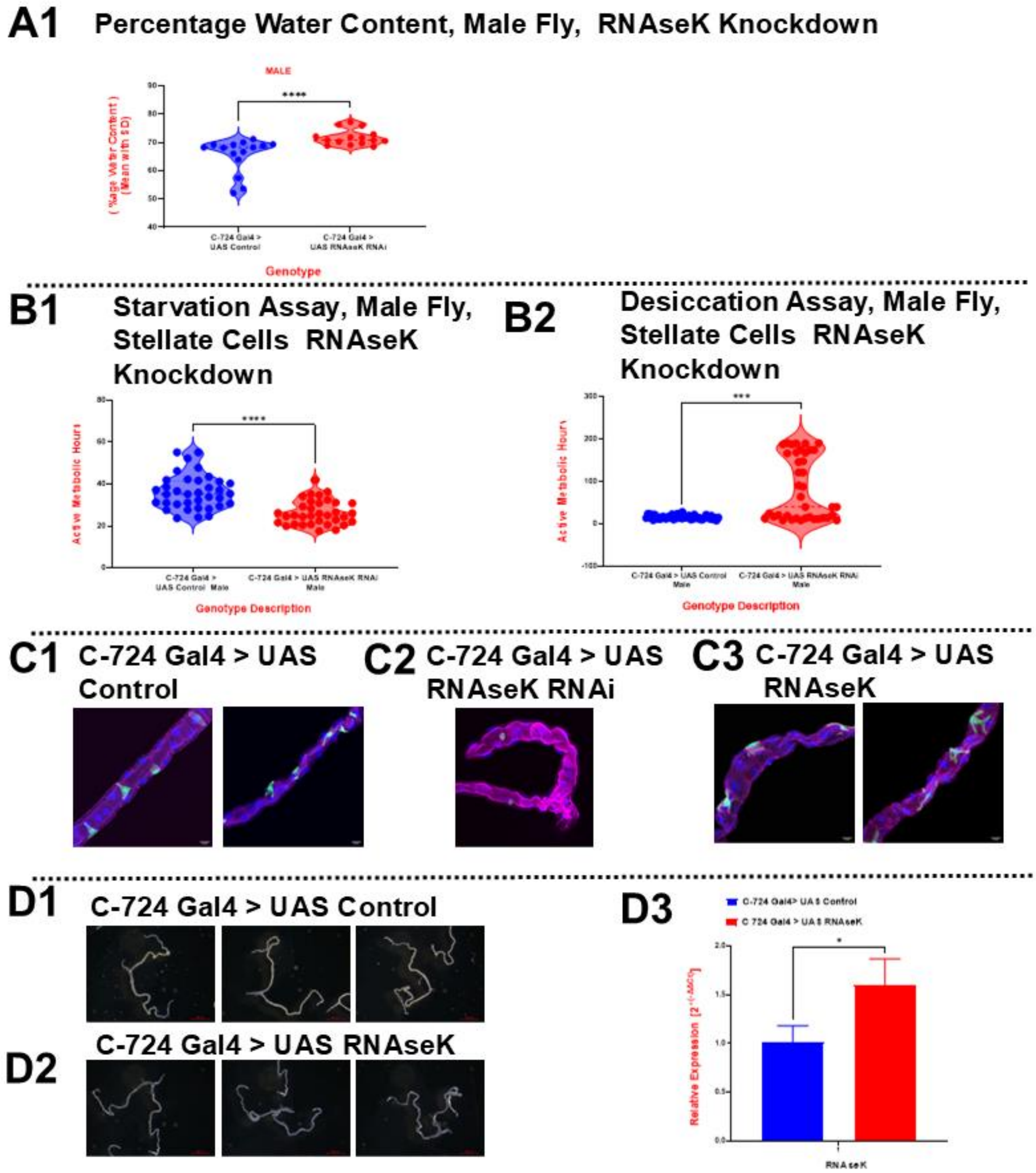

Fig. S10. Stellate-cell RNAseK manipulation alters water balance, stress tolerance, cell architecture and tubule pigmentation.

(A1) presents percentage body water content in 15-days old male fly with RNAseK RNAi in stellate cells, revealing a significant increase (****, $p \leq 0.0001$). (B1) and (B2) display results of starvation and desiccation assays showing that RNAseK knockdown in stellate cells reduces starvation resistance but enhances desiccation tolerance (****, $p \leq 0.0001$), (***, $p \leq 0.001$). (C1– C3) are projection images of tubules stained with Phalloidin (magenta), Dapi (blue), and mGFP (green) marking stellate cells; comparison indicates that RNAseK knockdown reduces stellate cell number and size (C2), while overexpression increases cell size (C3) relative to controls. Finally, tubule images in (D1) and (D2) show that RNAseK overexpression leads to loss of tubule pigmentation, and (D3) quantifies this overexpression, showing a significant increase in RNAseK expression (*, $p \leq 0.05$, Mann–Whitney test). Scale bars: 20µm (C1, C2, C3); 500µm (D1, D2).

Journal of Experimental Biology • Supplementary information

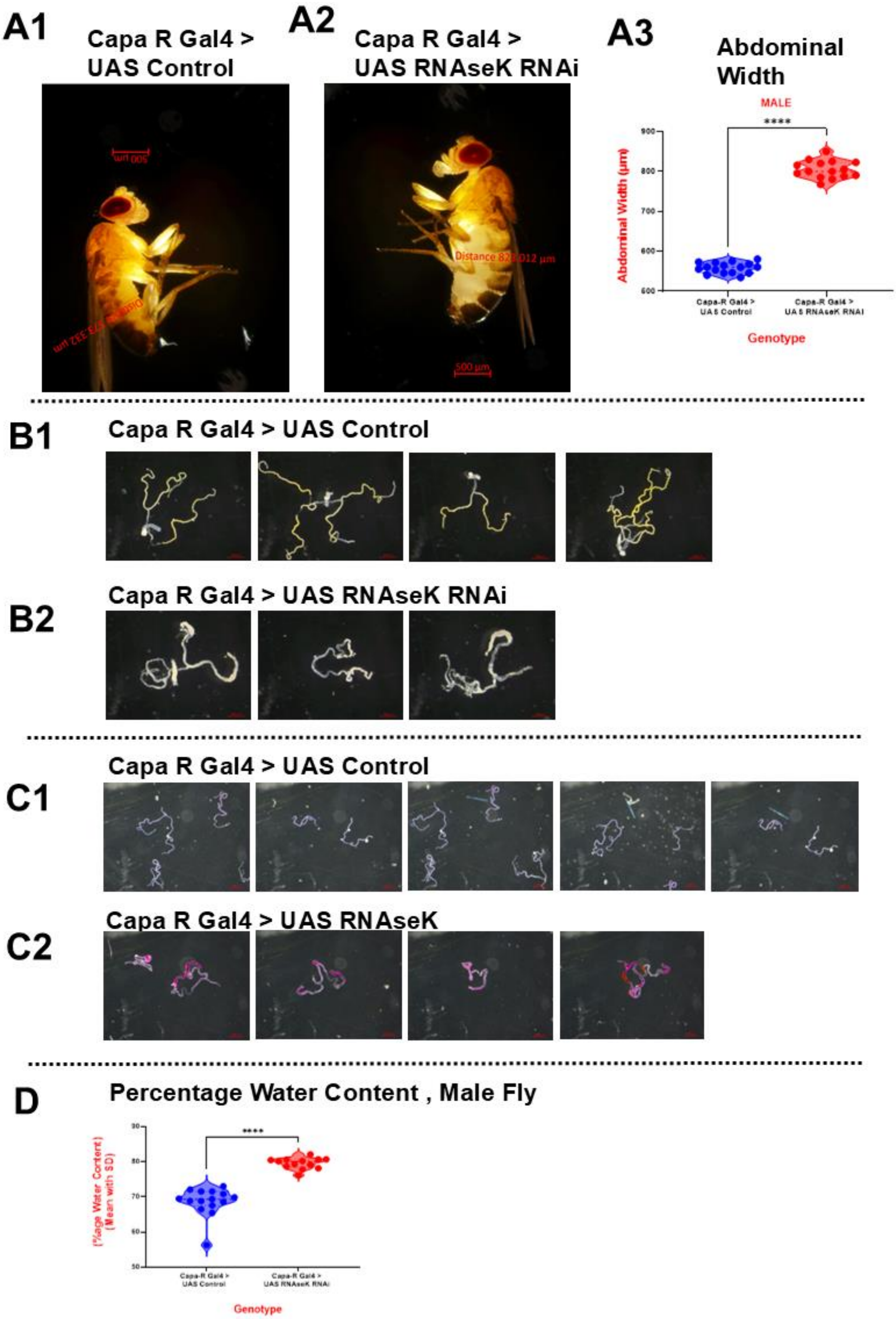
A1
Capa R Gal4 >
UAS Control
A2
Capa R Gal4 >
UAS RNAseK RNAi
A3
Abdominal
Width
MALE
****
Abdominal Width (µm)
900
800
700
600
500
Capa-R Gal4 > UAS Control
Capa-R Gal4 > UAS RNAseK RNAi
Genotype
500 µm
B1
Capa R Gal4 > UAS Control
B2
Capa R Gal4 > UAS RNAseK RNAi
C1
Capa R Gal4 > UAS Control
C2
Capa R Gal4 > UAS RNAseK
D
Percentage Water Content , Male Fly
****
(%age Water Content) (Mean with SD)
90
80
70
60
50
Capa-R Gal4 > UAS Control
Capa-R Gal4 > UAS RNAseK RNAi
Genotype

Fig. S11. Principal-cell RNAseK knockdown causes water retention and cystic, crystal-forming tubules.

(A1) shows a 9-days old control male fly, while (A2) depicts a fly of the same age group with RNAseK knockdown in principal cells exhibiting a visibly swollen abdomen. (A3) presents quantitative analysis confirming a significant increase in abdominal width in RNAseK RNAi flies (****, $p \leq 0.0001$, Mann–Whitney test). (B1) and (B2) display Malpighian tubules from control and RNAseK RNAi flies, respectively, both aged 9-days, revealing altered tubule morphology with cysts and loss of pigmentation under RNAi conditions. (C1) shows control tubules stained with Alizarin Red, whereas (C2) shows RNAseK RNAi tubules in Principal cells with prominent red stained crystalline deposits, indicating abnormal accumulation within the lumen. (D) shows a significant increase in body water content in RNAseK RNAi flies compared with controls (****, $p \leq 0.0001$). Scale bars: 500µm

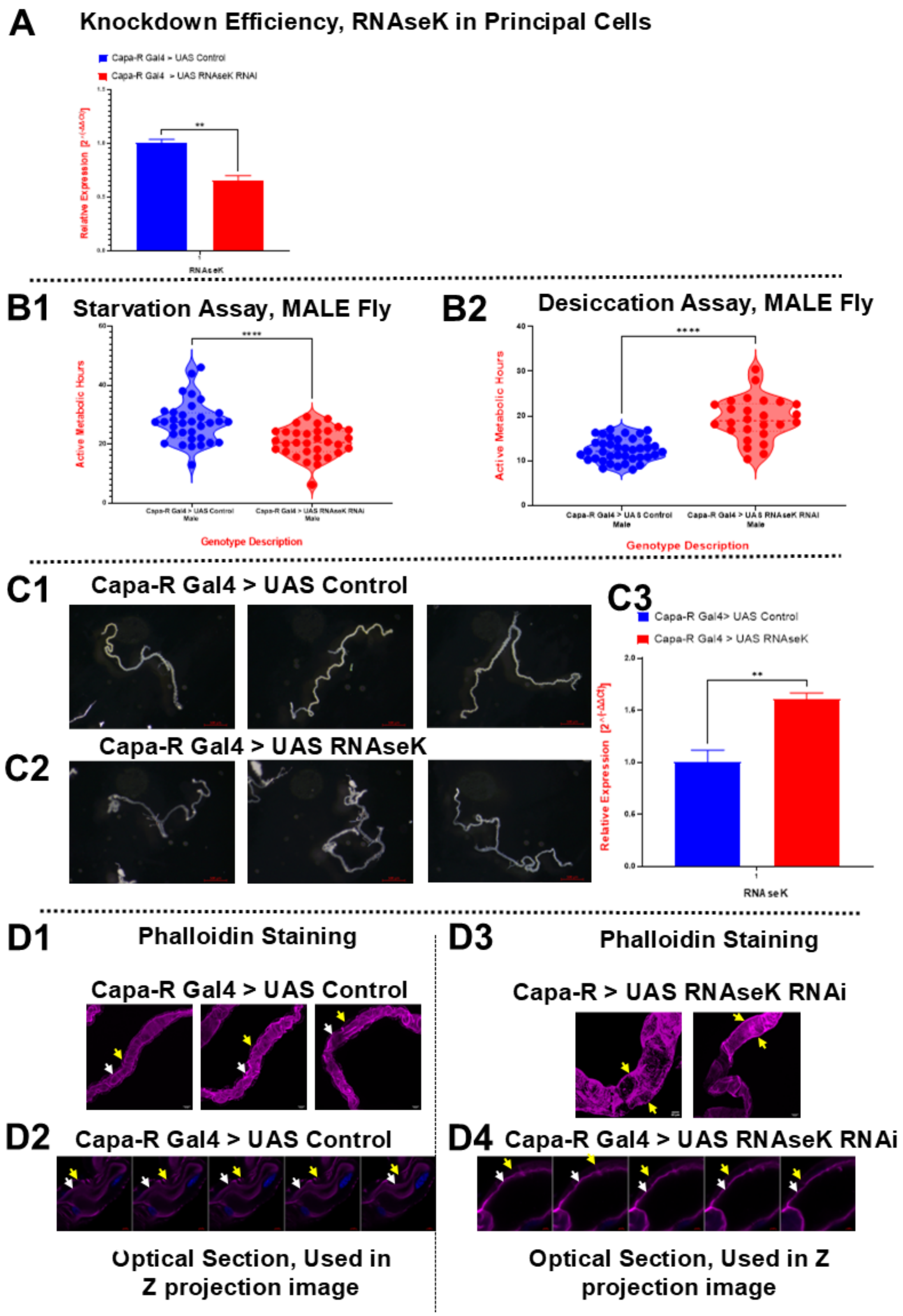

Journal of Experimental Biology • Supplementary information

Fig. S12. Principal-cell RNAseK manipulation disrupts stress tolerance, tubule pigmentation and apical–basal organisation.

(A) represents statistically significant RNAseK knockdown in Principal cells of tubule (**, $p \leq 0.01$). (B1) and (B2) display starvation and desiccation assays in male fly demonstrating that RNAseK knockdown in principal cells reduces starvation resistance but enhances tolerance to desiccation stress (****, $p \leq 0.0001$ for both). (C1) presents control tubules expressing Capa-R Gal4, while (C2) shows tubules overexpressing RNAseK exhibiting complete loss of pigmentation and a transparent phenotype but no cyst; (C3) confirms this by showing a significant increase in RNAseK expression (**, $p \leq 0.01$, Mann–Whitney test). (D1) shows additional projection images of the control tubule beyond those in the main image, with the white arrow indicating the apical layer and the yellow arrow marking the basal layer. (D2) presents single-section images used to generate the z-projections shown in the main image of control tubules stained with phalloidin, where white arrows marks uniform brush-border apical surfaces and yellow arrows indicate intact basal layers along the tubule length. In contrast, (D3) displays additional projection images from the main RNAseK RNAi tubule, where the yellow arrow points to fused apical and basal layers. (D4) shows individual sections of main images used to create the projection in the RNAseK RNAi principal cell tubules, showing the apical brush-border structure is disrupted and indistinct (white arrow), basal layers (yellow arrow). Scale bars: 500µm (C1, C2); 20µm (D1,D3); 5µm (D2, D4).

Journal of Experimental Biology • Supplementary information

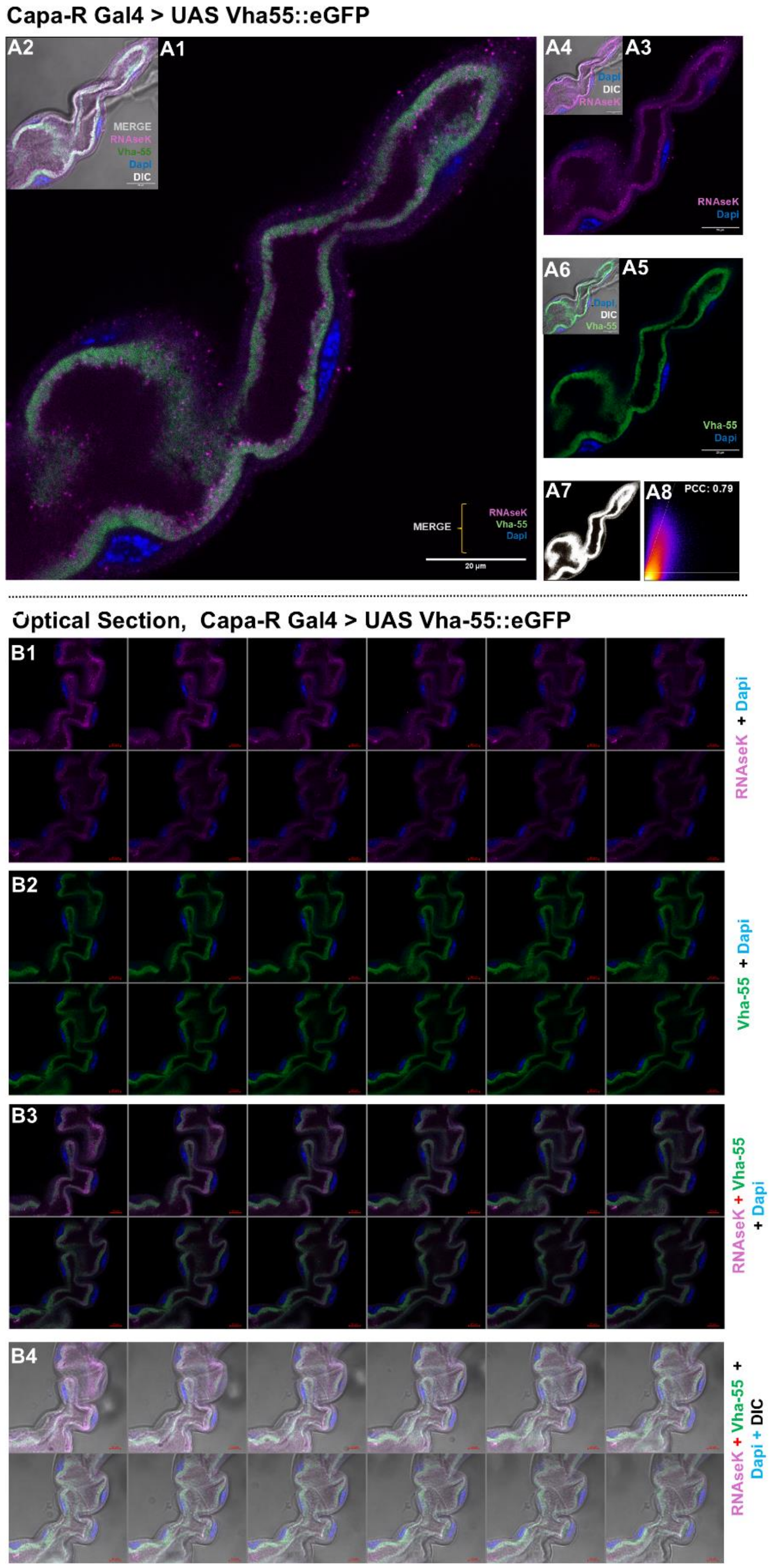
Capa-R Gal4 > UAS Vha55::eGFP
A2
A1
MERGE
RNAseK
Vha-55
Dapi
DIC
A4
A3
Dapi
DIC
RNAseK
RNAseK
Dapi
A6
A5
Dapi
DIC
Vha-55
Vha-55
Dapi
A7
A8
PCC: 0.79
MERGE
RNAseK
Vha-55
Dapi
20 µm
Optical Section, Capa-R Gal4 > UAS Vha-55::eGFP
B1
RNAseK + Dapi
B2
Vha-55 + Dapi
B3
RNAseK + Vha-55 + Dapi
B4
RNAseK + Vha-55 + Dapi + DIC

Fig. S13. RNAseK colocalises with Vha55 at the apical membrane of principal cells in Malpighian tubules.

(A1) shows an additional confocal image complementing that in the main text, acquired from 1 µm optical section thickness using identical imaging parameters across all channels. The merged image demonstrates the precise colocalization of RNAseK (magenta, immunostaining) with Vha55::eGFP (green) along the apical membrane of principal cells in Malpighian tubules overexpressing Vha55, while Dapi (blue) marks nuclei. (A2) overlays the same with DIC to confirm spatial apical distribution within tissue. (A3) and (A4) show single and DIC merged channels highlighting the apical localization of RNAseK, similarly (A5) and (A6) illustrate Vha55::eGFP expression and its apical correspondence. (A7) represents the white colocalization zones, and (A8) presents scatter plot confirming strong spatial molecular colocalization of RNAseK and Vha55. (Panels B1–B4) show 1-µm-thick optical sections that complement the main image findings, taken from the same tissue of optical sections. These confirm the overlap of RNAseK (B1) and Vha55 (B2) for each individual section, with merged DIC and fluorescence images delineating their apical localization in each section (B4). Scale bars: 20µm (A1-A7); 10µm (B1-B4).

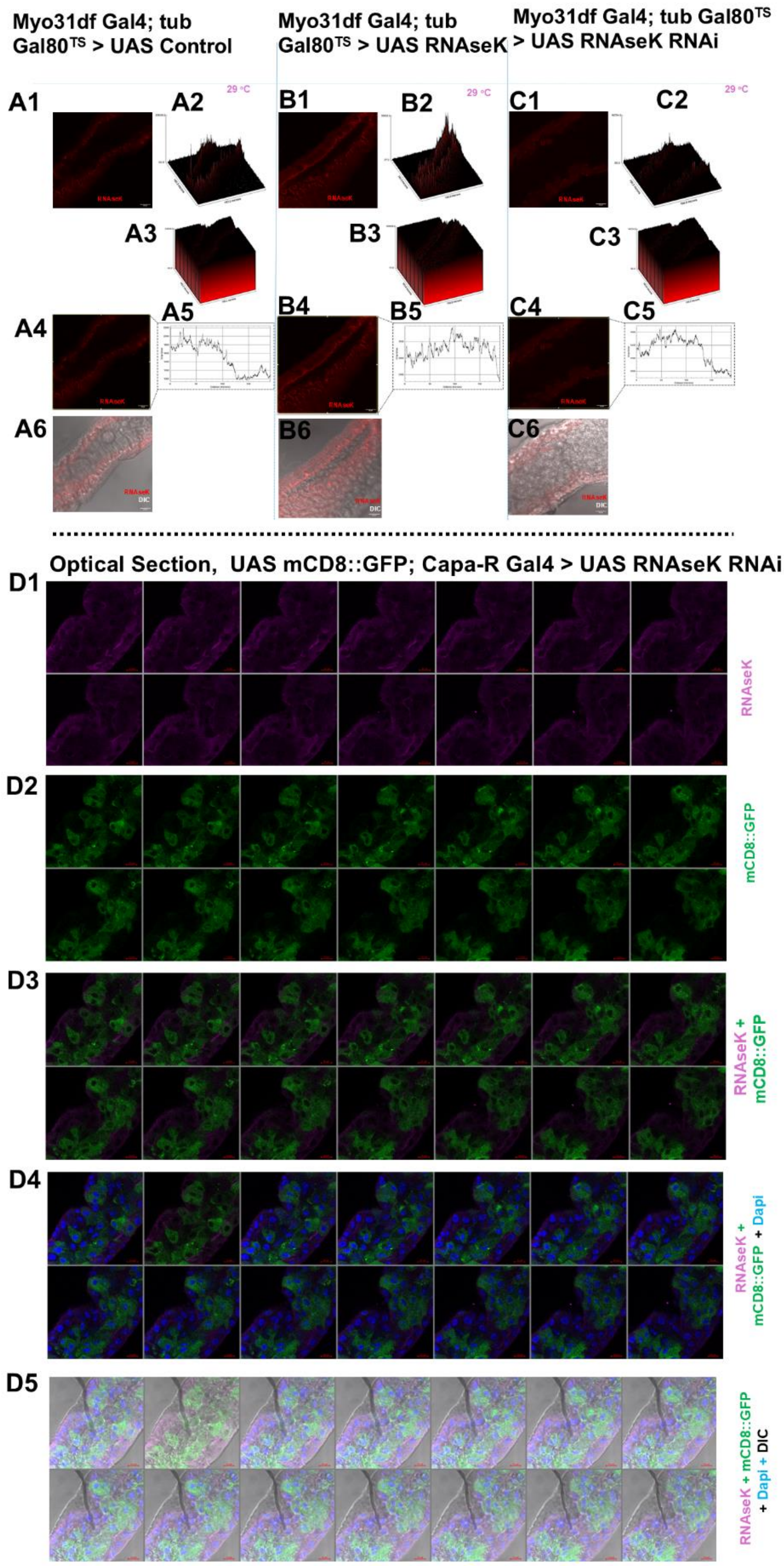

Journal of Experimental Biology • Supplementary information

Fig. S14. RNAseK expression changes validate antibody specificity and reveal apical-to-basal mislocalisation after knockdown.

Panels (A–C) display confocal projection analyses from 16 optical sections for each group of middle midgut region from female flies raised at 18 °C until eclosion and shifted to 29 °C after that, for 9-days to ensure stable expression. (A1–A6) display confocal images of the control genotype gut. (A1) shows the z-projection image. (A2) presents the surface plot (Wireframe, Axis, Shade, Max Y value is 23669). To provide another viewing angle for better clarity, (A3) shows the surface plot with a lighter source background. (A4) shows the complete rectangular surface containing the microscopic view selected for calculating the intensity profile in (A5), which is the grayscale image, maximum gray value 2200. (A6) represents the DIC image merged with RNAseK expression showing its spatial apical distribution. B1–B6 represents the RNAseK overexpression plots. (B1) shows the z-projection image. (B2) presents the surface plot (Wireframe, Axis, Shade, Max Y value is 30858). Again to provide another viewing angle, (B3) shows the surface plot with a lighter source background for better clarity. (B4) shows the complete rectangular surface containing the tissue microscopic view selected for calculating the intensity profile in (B5), which is the grayscale image, maximum gray value 3600. (B6) represents the DIC image merged with RNAseK expression showing its spatial apical distribution. (C1–C6) represents the RNAseK overexpression plots. (C1) shows the z-projection image. (C2) presents the surface plot (Wireframe, Axis, Shade, Max Y value is 16734). Again to provide another viewing angle, (C3) shows the surface plot with a lighter source background for better clarity. (C4) shows the complete rectangular surface containing the tissue microscopic view selected for calculating the intensity profile in (C5), which is the grayscale image, maximum gray value 1700. (C6) represents the DIC image merged with RNAseK expression showing its spatial apical distribution. The intensity data confirm proportional fluorescence changes enhanced upon genetic overexpression and reduced upon genetic knockdown—validating the antibody specificity. (D1–D4) show optical sections of Malpighian tubules with genetically downregulated RNAseK in principal cells, complementing the findings shown in the main image of the tissue. These images illustrate the mislocalisation of RNAseK from the apical to the basal membrane. (D1) shows RNAseK expression. (D2) shows principal cells expressing GFP. (D3) shows the colocalisation of RNAseK and mCD8::GFP. (D4) shows optical sections displaying RNAseK, mCD8::GFP, and DAPI staining. (D5) shows composite optical sections with RNAseK, mCD8::GFP, DAPI, and DIC images, confirming that, upon genetic downregulation in the tissue, RNAseK is not limited to the apical section; it is dispersed from apical to basal regions in the tubule.

Table S1. *Drosophila* Transgenic Lines

**Transgenic Line (Full Genotype)**	**Category**	**Functional Purpose**	**Source (Collection / Citation)**
y v; P{y[+t7.7]=CaryP}attP2	Genetic Control	Standard background control for crosses involving transgene insertions for Chromosome 3.	Bloomington Stock Center (BL #36303)
y sc[] v sev; P{y[+t7.7] v[+t1.8]=TRiP.HMS01529}attP2/TM3, Sb	Effector (RNAi)	UAS-inducible line for targeted knockdown of RNAseK.	Bloomington Stock Center (BL #35779)
y w[]; PBac{y[+mDint2] w[+mC]=UAS-RNASEK.HA}VK00033	Effector (Overexpression)	UAS-inducible line for overexpression of HA-tagged RNAseK.	Bloomington Stock Center (BL #94316)
w; P{w[+mC]=PTT-GA}VhaSFD[G00259]/CyO	Hypomorphic Line	It provides a sensitized background for studying the VHA hypomorphic effect on pH partitioning and V-ATPase localization while maintaining organismal viability in the heterozygous state.	Bloomington Stock Center (BL #6840)
*P{w[+m]=sens-GAL4.B}1, y w[]**	Driver	Directs expression in salivary gland under the control of the senseless promoter.	Bloomington Stock Center (BL #42740)
Myo31df-GAL4 ; tub-Gal80TS	Conditional Driver	This line was employed for temporally controlled transgene induction specifically within the adult midgut. This system allowed us to bypass early developmental lethality and demonstrate that RNAseK is intrinsically required to maintain the intestinal paracellular barrier. Specifically, it enabled the visualization of localized F-actin collapse and the execution of midgut pH partitioning studies.	Adam Dobson Lab (University of Glasgow, Scotland, UK)

Da-GAL4 ; tub-Gal80TS	Conditional Driver	Utilized for temporally controlled transgene induction across all tissues. By employing temperature-sensitive Gal80, we bypass early developmental lethality to study the adult-specific requirements of *RNAseK* RNAi for visualizing tissue and assessing systemic homeostasis.	Ilan Davies Lab (University of Glasgow, Scotland, UK)
Irk-2-GAL4/Cyo ; UAS-GFP/TM6c	Driver / Marker	A hindgut driver introgressed with a GFP reporter. It was utilized for allowing for the visualization of localized tissue defects and the study of how RNAseK knockdown disrupts hindgut-specific pH regulation.	B. Denholm Lab (University of Edinburgh, Scotland, UK)
w; CapaR-GAL4	Driver	An enhancer-trap line that directs expression specifically in Principal (Type I) cells; it serves as a reporter for Capa Receptor (CapaR) expression within the Malpighian tubule.	In-house; described in Terhzaz et al. (2012)
w; UAS mCD8::GFP; CapaR-GAL4	Driver / Marker	Principal cell-specific Gal4 driver introgressed with membrane-tethered GFP used to visualize cell boundaries in principal cells.	In-house; described in Terhzaz et al. (2012)
C-724 GAL4	Driver	An enhancer-trap line that directs expression specifically in Stellate (Type II) cells; it serves as a reporter for a subset of endogenous *teashirt (tsh)* expression within the Malpighian tubule.	In-house; described in Sözen et al. (1997)
w; C-724 GAL4; UAS-mGFP	Driver / Marker	An introgressed stable line combining the Stellate-specific C-724 driver with a UAS-mGFP reporter to facilitate imaging of the Type II cell population.	In-house; described in Dornan et al. (2023)

Journal of Experimental Biology • Supplementary information
